# Polycyclic Aromatic Hydrocarbons (PAHs) in Freshwater Systems: A Comprehensive Review of Sources, Distribution, and Ecotoxicological Impacts

**DOI:** 10.3390/toxics13040321

**Published:** 2025-04-20

**Authors:** Pedro J. Berríos-Rolón, María C. Cotto, Francisco Márquez

**Affiliations:** Nanomaterials Research Group, Department of Natural Sciences and Technology, Division of Natural Sciences, Technology and Environment, Universidad Ana G. Méndez-Gurabo Campus, Gurabo, PR 00778, USA; berriosp1@uagm.edu

**Keywords:** polycyclic aromatic hydrocarbons, water pollution, ecotoxicology, environmental matrices, freshwater ecosystems

## Abstract

This comprehensive review offers new perspectives on the distribution, sources, and ecotoxicological impacts of polycyclic aromatic hydrocarbons (PAHs) in freshwater systems. Unlike previous reviews, this work integrates recent findings on PAH dynamics within environmental matrices and emphasizes spatiotemporal variability across geographic regions. It critically examines both anthropogenic and natural sources, as well as the physical, chemical, and biological mechanisms driving PAH transport and fate. Special attention is given to the ecotoxicological effects of PAHs on freshwater organisms, including bioaccumulation, endocrine disruption, and genotoxicity. Notably, this review identifies key knowledge gaps and proposes an interdisciplinary framework to assess ecological risk and guide effective monitoring and management strategies for the protection of freshwater ecosystems.

## 1. Introduction

### 1.1. Polycyclic Aromatic Hydrocarbons

Polycyclic aromatic hydrocarbons (PAHs) are semi-volatile organic pollutants composed of two or more fused aromatic rings. They are classified into low molecular weight PAHs (LMW-PAHs) (2–3 rings) and high molecular weight PAHs (HMW-PAHs) (4 or more rings), with the latter posing greater carcinogenic, mutagenic, and genotoxic risks due to their higher hydrophobicity and lipophilicity [1,2]. PAHs are derived from three main sources: pyrogenic, petrogenic, and biogenic processes, which result from either natural phenomena or human activities. Pyrogenic PAHs are the by-products of incomplete combustion and/or pyrolysis of organic matter (OM) under low or no oxygen conditions, such as fossil fuel burning, wildfires, volcanic activity, coal burning, and anthropogenic activities [3]. On the other hand, petrogenic PAHs result from the diagenesis of organic materials over geological timescales, typically associated with crude oil, petroleum products, and their derivatives [4,5,6]. Although less common, biogenic PAHs are synthesized by plants [7,8], bacteria, fungi, and phytoplankton in specific ecological niches without involving diagenesis processes [9,10]. These distinct sources of PAHs can be identified using diagnostic ratios and molecular markers, which are valuable tools for tracking PAH pollution in the environment [11,12,13].

**Figure 1:**
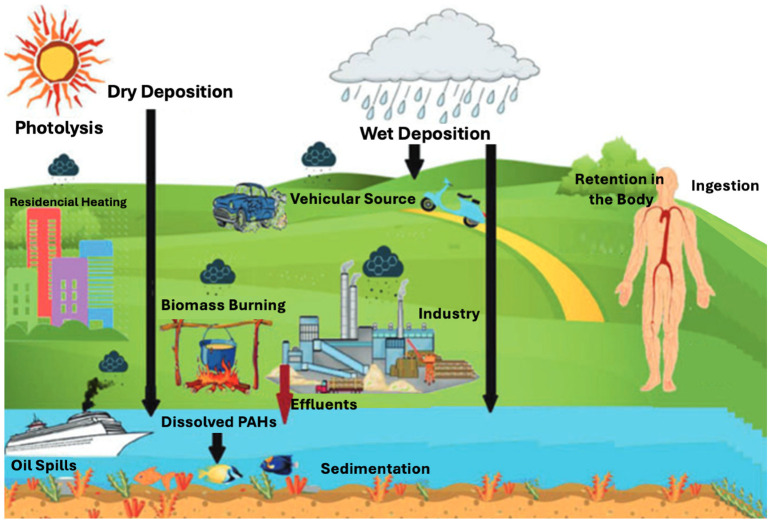
Distribution of PAHs in air, terrestrial, and aquatic environments. Reprinted with permission from ref. [17], Copyright 2019, Springer.

Upon formation, PAHs enter the atmosphere either in gaseous form or bound to particulate matter [14]. LMW-PAHs remain in the vapor phase, while HMW-PAHs adsorb to particulate matter, facilitating long-range transport (LRT) [15,16]. These atmospheric PAHs are subsequently deposited onto terrestrial and aquatic environments through wet and dry deposition processes [3]. Figure 1 illustrates the dynamics of PAHs in the environment, highlighting their mobility upon deposition on terrestrial surfaces [17], driven by runoff and weathering processes, and their subsequent transport into freshwater systems [18].

Globally, PAHs are a major concern due to their toxicity and widespread presence in air, water, and soil. In response, the United States Environmental Protection Agency (USEPA) designated 16 PAHs as priority pollutants due to their environmental prevalence [19] and associated human health risks [20,21]. Various international organizations, including the World Health Organization (WHO) [22], the Canadian Council of Ministers of the environment [23], and Greenpeace [24], have established monitoring frameworks and policies to address PAH contamination. Despite these efforts, mitigating PAH pollution remains a challenge, particularly in freshwater systems where their behavior is influenced by complex environmental interactions. Table S1 provides an overview of the physical and chemical properties of the 16 USEPA priority PAHs [25,26,27,28].

In aquatic environments, PAHs pose ecological and health risks to both aquatic organisms and humans [17]. Common pollution pathways include sewage discharges [29], industrial effluents [30], and runoff from urban [31,32] or agricultural areas [33]. In water, PAHs are distributed across dissolved phases, bound to OM, or adsorbed onto particulate matter or benthic sediments [34,35,36,37]. Their distribution within the water column is further influenced by the sediment–water partition coefficient [38], as their hydrophobic nature promotes adsorption onto suspended sediments, soil, and OM, contributing to their persistence and bioaccumulation in aquatic ecosystems [35,37].

PAHs exert toxic effects on a wide range of living organisms, including humans, animals, and microorganisms. Several PAHs are classified as genotoxic, mutagenic, carcinogenic, and teratogenic, thereby posing health risks to biological systems [39,40]. Their persistence is attributed to the dense π-electron system in their aromatic rings, which makes them resistant to nucleophilic attack [41]. HMW-PAHs are less volatile, more lipophilic, hydrophobic, environmentally persistent, and resistant to biodegradation [42,43]. These compounds readily bind to dissolved OM in sediments, accumulating in aquatic environments and increasing toxicity in benthic regions [36,44,45]. Additionally, rain runoff can transport soil-bound PAHs into water bodies, affecting flora, aquatic organisms, and food chains, ultimately posing direct risks to human health [46]. The lipophilic property of PAHs facilitates bioaccumulation across food webs, leading to human exposure [47,48,49]. Overall, the persistence and toxicity of PAHs in aquatic environments present significant risks to both human health and ecological systems.

### 1.2. Freshwater Systems

Freshwater systems cover only 2.5% of the Earth’s surface and are among the most vital resources for human consumption and biodiversity [50,51,52]. These systems include lakes, ponds, reservoirs, rivers, streams, groundwater aquifers, estuaries, and wetlands, collectively accounting for 0.8% of the Earth’s surface area [53,54,55]. Despite their importance, freshwater resources are under constant pressure, not only due to their geographical limitations but also from anthropogenic activities such as industrialization, agriculture, and urbanization, which contribute to the introduction of PAHs in these systems [54,56].

PAHs in freshwater systems present considerable risks to aquatic life, particularly to invertebrates, fish, and microorganisms. Their lipophilicity and hydrophobicity facilitate bioaccumulation, while their metabolisms can generate reactive intermediates, leading to oxidative stress, DNA damage, and endocrine disruption [43,57,58]. Even at low concentrations, PAHs negatively affect reproduction, growth, and survival in aquatic organisms, disrupting population dynamics and compromising ecosystem stability [45,59].

The occurrence, distribution, and fate of PAHs pose a global threat to freshwater ecosystems, regardless of geographical location [60,61,62]. The input of PAHs from point and non-point sources generates spatial and temporal heterogeneity in their distribution within these environments [63,64]. Through atmospheric deposition, surface runoff, and water discharges, PAHs enter water bodies where they either adsorb onto suspended sediments or persist dissolved in water [65]. Specifically, in lentic systems like lakes and wetlands, PAHs tend to accumulate in sediments due to slower water movement, whereas, in lotic systems such as rivers, they are transported over longer distances, promoting downstream pollution [66]. The resuspension of sediment-bound PAHs increases the exposure risk to aquatic organisms and facilitates LRT within watersheds, eventually reaching marine environments [67]. Although awareness of atmospheric PAHs has increased, significant gaps remain in understanding their transport, deposition, and dynamics within freshwater systems—particularly regarding their long-term ecological impacts [68].

### 1.3. Current Research Gaps and Emerging Perspectives in PAH Studies in Freshwater Systems

Despite significant progress in understanding the behavior and toxicity of PAHs in freshwater systems, key challenges remain unresolved. These include methodological inconsistencies [63,64], challenges in source identification and apportionment across spatial scales [69,70,71], and the resulting limitations in comparing findings or drawing generalizable conclusions. Additionally, the behavior of PAHs across environmental compartments—such as sediments, water, and biota—varies according to their physicochemical properties, further complicating efforts to predict their environmental fate and bioavailability [72,73,74,75]. While the toxicological mechanisms of PAHs in individual organisms are well-documented, their broader impacts at the community and ecosystem levels remain only partially understood. Understanding how PAHs affect trophic interactions and alter food web dynamics is essential to developing effective management and remediation strategies [76].

Numerous recent literature reviews have examined the environmental behavior and toxicological effects of PAHs across diverse matrices, including soil, sediments, air, and marine ecosystems [3,62,68,77,78,79,80,81,82]. However, most freshwater-specific reviews have focused on isolated components of the issue—such as sediment-associated contamination, species-specific toxicological responses, or individual ecosystem types—without integrating the complex interactions among PAH sources, transport pathways, environmental compartments, and ecological effects across diverse freshwater systems [45,46,59,79,81,83,84].

This review aims to provide a comprehensive analysis by critically synthesizing recent findings on PAH sources, environmental transport mechanisms, distribution patterns, and ecotoxicological impacts across various freshwater environments—including rivers, lakes, wetlands, and groundwater. Beyond compiling data, this work emphasizes spatial and temporal variability, ecosystem-level responses, and the influence of multiple stressors, such as climate change and co-contaminants. In doing so, it offers a comprehensive framework to interpret past findings and guide future monitoring, risk assessment, and management strategies. This review proposes an analysis by integrating ecological complexity and highlighting emerging research directions in the study of PAHs in freshwater systems.

## 2. Sources of PAHs in Freshwater Systems

PAHs enter freshwater systems through natural and anthropogenic processes. Source apportionment is commonly performed using techniques such as molecular diagnostic ratios, principal component analysis (PCA), and positive matrix factorization (PMF), which help differentiate between pyrogenic sources—such as fossil fuel combustion—and petrogenic sources, including crude oil or petroleum derivatives [85]. The emission and distribution of PAHs are influenced by a combination of geographic features, hydrological conditions, land use patterns, and human activities [69,70,71,86,87]. Furthermore, the physical and chemical properties of freshwater bodies, combined with climate variability, affect the behavior, transport, and accumulation of PAHs [88]. The unique characteristics of rivers, streams, lakes, wetlands, groundwater systems, and glaciers shape the contribution of different PAH sources and influence the resulting contamination dynamics in each environment [3,89,90].

### 2.1. Rivers

Rivers act as primary transport pathways for PAHs, carrying these pollutants from upstream sources to lakes, wetlands, or estuaries [91,92]. In the Buffalo River Estuary in South Africa, PAH concentrations in sediments reached up to 22,310 μg/kg and, in water, up to 206 μg/L, with diagnostic ratios indicating predominantly pyrogenic sources from automobiles, industrial effluents, and urban runoff [93]. Similarly, in the Bonny Estuary of the Niger Delta (Africa), sediment cores revealed total PAH concentrations ranging from 8699 to 22,528 μg/kg, with deeper layers enriched in pyrogenic PAHs and surface sediments showing elevated petrogenic levels linked to recent oil spills [94]. This pattern of mixed PAH sources is also observed in other rivers, such as the Niger Delta and the Amazon Basin, where pyrogenic and petrogenic PAHs have been identified—reaching up to 19,800 μg/kg in Niger Delta sediments and 163 ng/L in Amazon surface waters—originating from biomass burning, fossil fuel combustion, and oil-related activities [95,96]. In another study from Nigeria, surface water samples from the Ekulu River showed PAH concentrations as high as 3.17 mg/L, with a prevalence of HMW-PAHs derived from pyrogenic sources linked to combustion-related anthropogenic activities [97]. In the Middle East, the Euphrates River (Iraq) shows PAHs in water and sediments dominated by HMW species derived from petroleum product combustion, with carcinogenic PAHs comprising up to 55% of the total [98]. In contrast, surface sediments from the San Joaquin River in California (USA) revealed that surface layers were largely influenced by pyrogenic PAHs (~70%), reflecting inputs from recent urban landscape alterations, while deeper layers exhibited higher proportions of biogenic PAHs [99].

Rivers in China, such as the Yangtze and Haihe, are significantly impacted by combustion-related PAHs, especially from coal, coke, vehicle fuel, and biomass burning. In the Yangtze River Estuary, PAH concentrations in sediments ranged from 34.9 to 580.3 ng/g, with source apportionment revealing major contributions from vehicle emissions (38.4%) and coal combustion (15.8%) [100]. Sediments in the middle-lower Yangtze also act as secondary sources of LMW-PAHs through resuspension, while retaining HMW-PAHs in the lower reaches due to reduced sediment discharge caused by dam impoundment [74]. In the Haihe River, sewage discharge was identified as a major source of PAHs, significantly influencing their partitioning behavior between sediments and pore water; parent PAHs exhibited strong sorption capacity, with a logarithmic organic carbon normalized partition coefficient (log K_OC_) averaging 4.04 ± 0.80 [101]. Similarly, the River Benue in Nigeria exhibits mixed pyrogenic and petrogenic PAH sources, with sediment concentrations ranging from 55 to 382 μg/kg; source apportionment identified petrogenic burning (35%) and wood combustion (27%) as dominant contributors [102]. In Taiwan, sediment PAH concentrations across 30 major rivers reached up to 7.44 mg/kg, with pyrogenic sources prevailing and seasonal variations linked to wastewater discharge and combustion activities [103]. These findings emphasize the dual role of rivers as conduits and secondary sources of PAHs, with combustion-related emissions frequently dominating due to regional industrial activities and seasonal variability in land-use patterns.

### 2.2. Streams

Streams, especially those flowing through urban and forested landscapes, are strongly impacted by PAHs derived from combustion-related activities, primarily of pyrogenic origin. In China, sediment samples from urban streams in the Suzhou Industrial Park revealed total PAH concentrations ranging from 180 to 81,000 ng/g, with four-ring PAHs being the most abundant (42 ± 12%) and source apportionment pointing to coal and biomass combustion (61%) as the primary contributor, followed by vehicular emissions (18%) [104]. In the United States, a study across 10 urban watersheds found that streambed sediment PAH levels were significantly higher in regions where coal-tar-based pavement sealants were widely used—up to 54.3 mg/kg in the Northeast—underscoring pavement dust and sealed surfaces as major vectors of PAH transport to urban streams [105]. In Poland, sediment from retention tanks along the Oliwski and Strzyza streams showed PAH concentrations up to 20.4 mg/kg, with 4- and 5-ring PAHs predominating due to traffic emissions, heating systems, and vehicular abrasion, and presenting moderate- to high-risk levels to benthic organisms in certain locations [106]. These urban examples contrast with forested stream systems, where PAH contamination is more episodic. For instance, post-wildfire runoff in Portugal has been shown to increase PAH loading in streams due to soil erosion and ash transport following fire events [107]. Evidence from recent reviews indicates that wildfires are a diffuse but significant source of PAHs, mobilizing them into surface waters and sediments through post-fire runoff and other transport processes, with impacts depending on fire severity, vegetation type, and hydrological conditions [46,108]. While streams are influenced by both petrogenic and pyrogenic PAH sources, recent studies suggest that pyrogenic inputs—particularly those linked to combustion activities and urban expansion—tend to dominate, with some contributions originating from distant atmospheric sources [109,110].

### 2.3. Lakes

**Figure 2:**
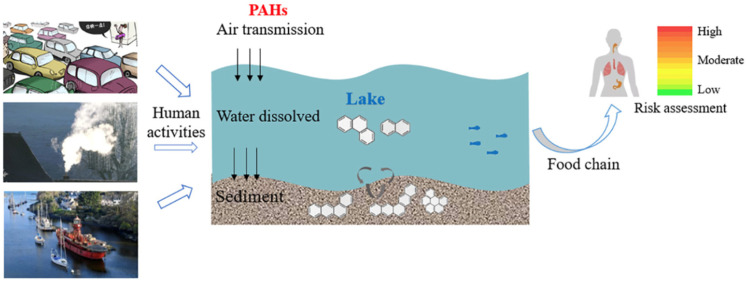
Sources, distribution, and transfer of PAHs through environmental compartments in freshwater lakes until human exposure is achieved. Reprinted with permission from ref. [115]; copyright 2019, Elsevier.

Lakes face challenges from a variety of PAH emission sources, particularly in urban areas, where petroleum, biomass, heavy oils, and natural gas consumption dominate [111]. Despite successful policies in several countries aimed at reducing point source pollution [112,113], non-point sources, such as atmospheric deposition and surface runoff, remain significant contributors to PAH contamination in lake systems [114]. Studies suggest that LMW-PAHs are found dissolved in the water column, and HMW-PAHs accumulate in lake sediments (Figure 2) [115,116]. In urban lakes, local non-point atmospheric deposition from pyrogenic activities often surpasses regional sources, as observed in Ontario Lakes in Canada [117]. Similarly, in Lake Baikal, PAHs primarily originate from biomass and fossil fuel combustion, with lighter PAHs concentrated in the water column and heavier ones sequestered in sediments [75]. Urbanization intensifies PAH concentrations, particularly in lakes exposed to high runoff from impervious surfaces, further amplifying the issue [111,118,119].

In contrast, remote lakes, which are less impacted by direct anthropogenic activities, often receive PAHs through atmospheric LRT and wildfires, which contribute to PAH deposition [46,120,121]. The rise in human activity, Gross Domestic Product, and population in lake catchment areas has also led to increased PAH levels, primarily from coal combustion and other pyrogenic sources [122,123,124]. Overall, PAH pollution in lakes results from both urbanization and natural processes, with urban lakes primarily affected by human activities, while the impact on remote lakes largely stems from natural phenomena, such as wildfires. These variations in source inputs and environmental dynamics between urban and remote lakes align with patterns observed across other freshwater systems worldwide. A comparative summary of PAH contamination, primary sources, and associated ecological effects reported in selected riverine and lacustrine systems is presented in Table 1, illustrating spatial variability and common risk factors across freshwater environments.

### 2.4. Wetlands

**Figure 3:**
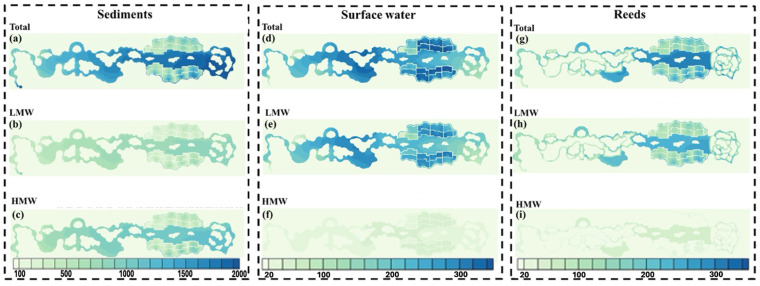
Distribution of total LMW-PAHs and HMW-PAHs in sediments, surface water, and reeds in a constructed wetland that receives water from a wastewater treatment plant. Simulated distribution results of sediments (**a**–**c**), surface water (**d**–**f**) and reeds (**g**–**i**) for Total PAHs. Reprinted with permission from ref. [140]; copyright 2021, Elsevier.

**Figure 4:**
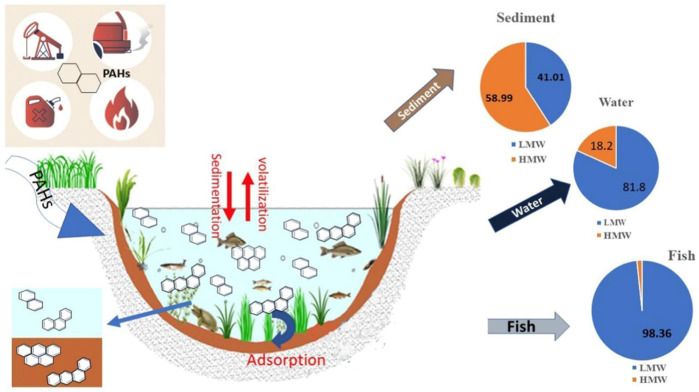
PAH sources and distribution in environmental matrices of a freshwater wetland. Reprinted with permission from ref. [141]; copyright 2023, Elsevier.

Beyond lakes, freshwater wetlands also represent critical and vulnerable systems affected by diverse PAH sources. Freshwater wetlands are highly productive ecosystems that are particularly vulnerable to PAH pollution from various sources [19,131,132]. These ecosystems, recognized for their vital ecological functions, are increasingly degraded by oil exploitation and human disturbances [133,134]. The sources of PAHs in wetlands vary by region and proximity to human activities. For instance, pollution in Iran’s Anzali Wetland is attributed to wastewater discharge, tourism, and oil-related activities [135,136,137], whereas China’s Northeastern wetlands experience contamination primarily from coal burning and vehicle emissions [138]. Seasonal variations also play a significant role, such as the predominance of petrogenic sources during the wet season in Wang Lake Wetland, China [139]. Furthermore, constructed and urban wetlands, essential for water treatment, also face PAH contamination from wastewater discharge and fossil fuel combustion, with PAH distribution strongly influenced by molecular weight (Figure 3) [140]. In wetlands, PAHs in the water column are often derived from petroleum sources, while sediment-bound PAHs are typically pyrogenic, highlighting the complex distribution of PAHs in these environments (Figure 4) [141].

In Ramsar-designated wetlands and other protected areas, mixed sources of PAHs, especially from oil-related activities, present ongoing challenges [142]. Wetlands near oil extraction sites, such as Shadegan Wetland in Iran and Hoor-Al-Azim Wetland in Lower Mesopotamia, are often contaminated by both pyrogenic and petrogenic sources, with oil spills and combustion-related emissions being primary contributors [19,143,144]. Lastly, peatlands, marshes, and bogs, such as those in Northeast China and Iraq’s Al-Hammar marshes, serve as environmental archives, reflecting historical pollution from both anthropogenic and natural sources, including wildfires and industrial activities [145,146,147]. Freshwater wetlands, critical for their ecological functions, face complex challenges for PAH pollution derived from mixed sources.

### 2.5. Groundwater

Groundwater, as a primary freshwater source for domestic, agricultural, and industrial purposes, is increasingly contaminated with PAHs introduced through diverse pathways [148,149]. PAHs leach from soils into aquifers or are transported via surface runoff, often impacting both surface and groundwater systems [68]. The strong hydrological connectivity between surface water and groundwater facilitates the migration of PAHs, particularly in closely linked systems [86,150]. Due to their higher mobility, LMW-PAHs can migrate more readily in soil, resulting in relatively uniform PAH concentrations across interconnected water systems [151].

In the Yellow River Delta, China, pyrogenic PAHs from petroleum combustion dominate groundwater pollution, entering aquifers primarily through surface water infiltration [150]. Similarly, in karst terrains, the rapid vertical migration of PAHs is enabled by geological features like conduits and sinkholes, transporting contaminants from the combustion of grass, wood, and coal [86,152,153]. Local factors within the river’s catchment area significantly influence PAH sources in such environments [151,154].

Pyrogenic sources are a primary contributor to PAH contamination in groundwater, often infiltrating aquifers through soil leaching and various transport mechanisms [155]. Agricultural practices, such as long-term irrigation with wastewater or reclaimed water, are the main source of groundwater PAHs in farming areas. Wildfires also impact groundwater quality by introducing pyrogenic PAHs during post-wildfire events, particularly in areas connected to public water supplies, resulting in risks to human health [156]. Furthermore, industrial activities near abandoned complexes and oil-producing regions contribute to both pyrogenic and petrogenic PAHs, resulting in mixed-source profiles due to vertical migration over extended periods of time [157,158]. The complex and diverse sources of PAH pollution in groundwater underscore the need to address both localized and long-term contamination pathways.

Economic development and changing anthropogenic activities shape the evolution of PAH emission sources. In the groundwater of karstic regions in Southwest China, a shift from coal combustion to vehicular emissions over a decade has sustained a predominantly pyrogenic PAH profile [159]. Geological factors, such as complex lithology and hydrostratigraphic systems, further influence PAH origins in groundwater, with carbon and fuel combustion serving as major pollution sources in these environments [160]. Even in oil fields such as the upper Brahmaputra Valley of India, surface combustion activities dominate groundwater contamination, surpassing petrogenic contributions from oil spills [155]. Despite regional variations, pyrogenic PAHs consistently represent a significant and persistent source of groundwater pollution.

### 2.6. Glaciers

Recent research highlights the sources of PAHs in various glacial freshwater environments. Attribution ratios in Arctic freshwater systems suggest that PAHs predominantly originate from atmospheric deposition and combustion-related sources [68]. Fresh meltwater samples from the eastern Tibetan Glacier show PAHs associated with incomplete coal combustion and coking that are deposited via LRT and are also affected by local environmental conditions [161]. These melting glaciers act as secondary sources of PAHs, leading to elevated concentrations in sediment cores of proglacial lakes and glacial-fed streams, establishing PAHs as the most dominant of the persistent organic pollutants in the Arctic environment [162]. This retention is facilitated by the OM content in proglacial soils, which promotes the accumulation of PAHs in these environments [163].

In the case of the Kongsfjorden Glacier in Norway, PAH concentrations decline with increasing distance from the glacier, with local petrogenic combustion processes, atmospheric LRT, and historical coal mining identified as key sources [164]. Additionally, dissolved PAHs in the Kongsfjorden Glacier meltwater further indicate major contributions from grass, wood, and coal combustion, with their distribution influenced by ocean currents and glacier runoff [165].

In Antarctica, the most remote continent and holder of the largest freshwater reservoir, PAH sources have been studied to better understand their dynamics in glacial environments. Reports suggest that Antarctic waters receive a mix of locally consumed fuel combustion and globally transported PAHs via LRT [166]. On King George Island, PAHs accumulate in terrestrial soils, primarily linked to electricity generators and light-duty gasoline consumption associated with scientific research and tourism activities [167]. However, terrestrial PAH pollution levels on King George Island are significantly lower than in other parts of the world [168]. Reducing fossil fuel consumption in these regions is recommended to mitigate PAH emissions and minimize their impact on Arctic and Antarctic glacial freshwater environments.

Overall, the presence of PAHs in freshwater systems—including lakes, rivers, streams, groundwater, wetlands, and glaciers—is shaped by diverse emission sources (see Table S2). Lakes face significant challenges from both point and non-point sources, with atmospheric deposition and surface runoff being primary contributors. Rivers act as major transport pathways for PAHs, reflecting mixed emission sources, while wetlands, groundwater, and glacial freshwater systems exhibit distinct patterns of PAH contamination. These patterns are supported by numerous case studies conducted across Asia, Africa, Europe, and the Americas [19,74,86,91,92,93,97,98,102,103,104,106,107,109,110,111,119,120,121,135,140,141,142,143,146,150,156,157,158,159,160,161,164,165,166,167,169,170,171,172,173,174,175,176,177,178,179,180,181]. These findings highlight the critical need for integrated management, robust monitoring, focused research, and effective policy strategies to address PAH pollution across freshwater systems.

## 3. Distribution of PAHs in Freshwater Systems

The distribution of PAHs in freshwater systems is influenced by various transport, mechanisms, environmental factors, and interactions with environmental matrices. Understanding these processes is essential for addressing the environmental distribution and fate of PAHs in these systems. This section explores the primary transport mechanisms and environmental factors influencing the distribution of PAHs, emphasizing their interactions with various environmental matrices in freshwater systems.

### 3.1. Transport Mechanisms

Polycyclic aromatic hydrocarbons (PAHs) reach freshwater systems through a range of environmental pathways, including atmospheric deposition, surface runoff, riverine transport, groundwater infiltration, sediment resuspension, and biological activity. These inputs are shaped by both local and distant anthropogenic sources—particularly combustion-related emissions—and modulated by climatic and land-use dynamics [61,182,183,184]. Once PAHs enter aquatic systems, their transport and eventual fate are governed by a combination of physical, chemical, and biological mechanisms that determine their partitioning, mobility, and persistence [185,186]. Understanding these mechanisms is essential to accurately assess the spatial and temporal distribution of PAHs, predict exposure scenarios, and inform risk assessments and management strategies. The following subsections explore each of these transport pathways in detail, emphasizing how system-specific factors such as flow regimes, sediment composition, and water chemistry influence PAH movement and accumulation in freshwater environments.

#### 3.1.1. Physical Transport Mechanisms

PAHs are physically transported in freshwater systems through advection, diffusion, and sediment dynamics, all of which are shaped by hydrological and environmental conditions. Among these, advection—the transport of pollutants via flowing water—is a predominant mechanism in lotic systems [74,187,188,189]. This process enables the downstream redistribution of PAHs from both local and distant sources. In the Yangtze River (China), Zhao et al. [74] demonstrated that water runoff from megacities—driven by coal and coke combustion—served as a major advective input, with the river current transporting PAHs downstream, while reduced sediment discharge from upstream dams increased their retention in lower reaches. Modeling of the Yangtze River Delta under future land-use scenarios further confirmed that advection is the principal mechanism governing PAH redistribution across environmental compartments [190]. In Dianshan Lake, a shallow urban system, Du et al. [189] found that atmospheric advection was the main source of PAHs, with hydrodynamic movement driving their transfer from water to sediment. In another report, in Lake Qinghai (China), PAH concentrations were highest at sediment sites near river inlets, supporting the role of river inflows as advective carriers of local emissions [188]. On the other hand, in groundwater systems, advective flow accelerated by irrigation pumping, significantly increased the subsurface mobility of low to medium molecular weight PAHs [191]. Additionally, in the Columbia River Estuary (USA), hydrodynamic trapping processes enhanced the downstream transport and retention of combustion-derived PAHs, even at distances far from original inputs [192]. Collectively, these studies highlight advection as a system-specific but ubiquitous mechanism that mobilizes PAHs across spatial gradients in both lotic and lentic freshwater environments.

Diffusive transport refers to the passive movement of PAH molecules from regions of high to low concentration until equilibrium is achieved. This process is particularly relevant in low-flow freshwater environments—such as lakes and wetlands—where water circulation is minimal and vertical gradients persist. For instance, in Panguipulli Lake (Chile), net volatilization of LMW-PAHs and deposition of heavier congeners were observed across the air–water interface, demonstrating how hydrophobicity and hydrodynamic stagnation influence the direction and magnitude of diffusive fluxes [193]. In the Willamette River (USA), paired samplers revealed that sediments acted as a long-term source of 4- and 5-ring PAHs, diffusing into the water column and volatilizing into the atmosphere despite prior remediation efforts [194]. In Dianchi Lake (China), model simulations showed that diffusion, alongside degradation, was a dominant removal mechanism for PAHs from sediments, counteracting accumulation from atmospheric deposition and waterborne inputs [195]. Likewise, observations across the Yangtze River Delta (China) indicated that PAHs such as naphthalene (NaP) and benzo[a]pyrene (BaP) were subject to resuspension and diffusion at sediment–water interfaces, with transport direction driven by compound-specific properties and local hydrological conditions [196]. In Poyang Lake (China), seasonal dry–wet cycles were found to regulate sediment-to-water PAH exchanges, with LMW congeners volatilizing during periods when sediments were exposed to air, and HMW compounds showing varied flux directions when submerged under water [197]. Altogether, these studies highlight diffusion as a dynamic and compound-specific process that governs the long-term distribution, bioavailability, and environmental persistence of PAHs in stagnant or weakly mixed freshwater systems.

**Figure 5:**
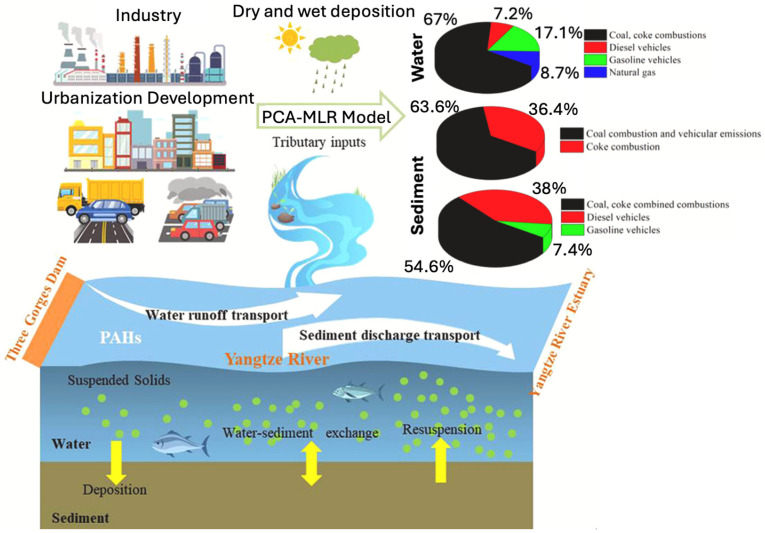
Water runoff from megacities, driven by coal and coke combustion sources, contributes to the transport and increased catchment retention of PAHs along the Yangtze River, China. Reprinted with permission from ref. [74]; copyright 2021, Elsevier.

The hydrophobic nature of PAHs facilitates their attachment to suspended particulate matter (SPM) and sediments, enhancing their persistence and transport in freshwater environments [198]. SPM serves as a mobile phase that carries PAHs across aquatic systems before settling through deposition, while sediments function as temporary reservoirs [122]. However, these reservoirs are not static and resuspension events triggered by storms, flooding, or human activities can remobilize sediment-bound PAHs into the water column, promoting downstream transport and increasing their bioavailability [123,199]. The extent and efficiency of PAH partitioning are influenced by multiple factors, including particle size, organic carbon (OC) content, hydrodynamics, and sediment characteristics [200,201]. Fine particles, in particular, remain suspended longer after resuspension events, allowing PAHs to be transported farther from their original source areas [200]. In contrast, sediments rich in OC tend to exhibit stronger PAH sorption and retention capabilities, reinforcing their role as long-term sinks [201]. Over time, sediments act as dynamic reservoirs—both absorbing and re-releasing PAHs—thus functioning as both sinks and secondary sources in rivers [36], lakes [202], and wetlands [203]. Figure 5 illustrates this dual role in the Yangtze River (China), where PAH transport is shaped by runoff, sediment discharge, deposition, and resuspension under the influence of urbanization and industrial emissions [74]. Overall, sediment dynamics critically shape the environmental fate of PAHs, determining whether these compounds remain stored or become remobilized under changing environmental conditions.

The physical transport of PAHs in freshwater systems is strongly modulated by hydrological factors, which govern their mobilization, redistribution, and persistence across diverse aquatic environments. Rainfall, for instance, facilitates the wash of PAHs from urban and industrial surfaces into water bodies, particularly during intense storm events that generate high surface runoff and sediment discharge [204]. In systems with seasonal hydrological variation, dry-season conditions with lower dilution capacity led to elevated PAH concentrations in water, while intense wet-season rainfall increased SPM, enhancing PAH sorption and downstream transport [205]. Similarly, flooding events, such as Hurricane Harvey in Houston, Texas (USA), caused large-scale PAH redistribution into Galveston Bay, with peak concentrations and biological responses observed shortly after flood onset [206]. Resuspension of contaminated sediments during floods has also been observed in the Niger Delta (Nigeria), intensifying ecological and health risks due to high levels of HMW-PAHs [79]. Seasonal hydrological changes further influence phase exchange processes; in tide-dominated estuaries, temperature and marine currents modulate PAH diffusion across sediment–water interfaces, with source contributions varying by season [116]. In major rivers such as the Yangtze River (China), temperature and discharge variations during the wet season favor the accumulation of HMW-PAHs, driven by increased flow and suspended solids [207]. On the other hand, in lentic systems, such as the Shuikou Dam (China), impoundment reduces flow and increases PAH retention, while wet-season surface runoff elevates PAH concentrations downstream of industrial zones [208]. Karst systems present unique hydrological pathways, where PAHs infiltrate rapidly through fissures and conduits, promoting swift subsurface transport from surface sources to spring-fed waters [152,153]. Finally, hydrogeological conditions—such as permeability, vadose zone thickness, and water table fluctuations—determine PAH distribution in aquifers, with variations observed across regions like Du’an, Lanzhou, and Golmud (China) [176]. In summary, hydrological processes such as rainfall, flooding, temperature shifts, and groundwater flow directly influence how PAHs are transported and where they accumulate in freshwater environments.

**Figure 6:**
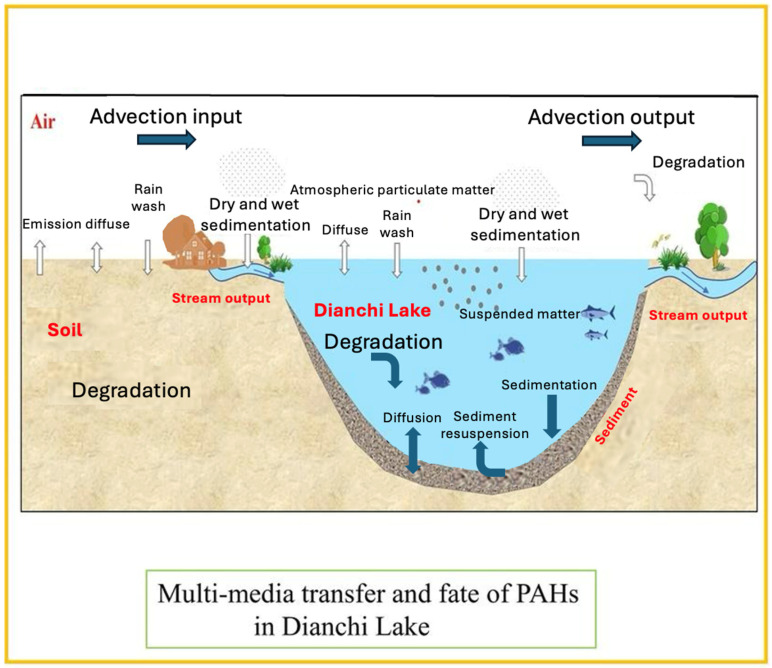
Multimedia transfer of PAHs in Dianchi Lake, China, illustrating advective, diffusive, and sediment transport mechanisms. The diagram shows the pathways of PAH movement through air, water, and sediment compartments, emphasizing advective inflows and outflows, diffusion dynamics, degradation processes, and interactions at the sediment–water interface. Reprinted with permission from ref. [195]; copyright 2024, Elsevier.

As detailed throughout this subsection, the physical transport of PAHs in freshwater systems is governed by three interrelated mechanisms—advection, diffusion, and sediment dynamics—and is strongly modulated by hydrological factors such as rainfall, flow regime, and seasonal variations. Advective transport, driven by river flow, groundwater movement, and internal circulation in lentic environments, facilitates the broad dispersal of PAHs across spatial gradients. Diffusion becomes particularly relevant in stagnant or low-flow zones, where the physicochemical properties of individual PAHs and prevailing environmental conditions control their gradual redistribution across phases. Sediment dynamics, including deposition and resuspension, mediate the long-term fate of PAHs by acting as both sinks and secondary sources, with their behavior influenced by sediment composition, organic content, and external hydrological forces. These mechanisms do not operate in isolation; rather, they interact within the unique hydrological frameworks of rivers, lakes, and wetlands, shaping the spatial and temporal dynamics of PAH transport. Figure 6 illustrates this complexity in Dianchi Lake (China), where model simulations have enabled the quantification of PAH fluxes across compartments, highlighting how different processes such as advection, diffusion, and sedimentation collectively determine the fate of pollutants in freshwater systems.

#### 3.1.2. Chemical Transport Mechanisms

The transport and distribution of PAHs in freshwater systems are influenced by chemical processes such as sorption and desorption, both of which are affected by environmental and chemical conditions. Sorption occurs when PAHs adhere to sediments or suspended matter, a process governed by molecular hydrophobicity, molecular weight, and steric factors [209,210]. Skic et al. [209] demonstrated that sorption in bottom sediments is facilitated by steric hindrance and the presence of negatively dissociating functional groups, especially for HMW-PAHs, with finer pores (<5 μm) serving as preferential sequestration sites. OM composition and structure also play a dominant role, as shown by Huang et al. [211], who reported that the heterogeneity of sediment OM—including humic substances, black carbon, and kerogen—results in nonlinear sorption behaviors such as hysteresis and slow desorption kinetics. Dissolved and particulate OC enhance PAH affinity across colloidal, dissolved, and particulate phases in underground river systems [44,212]. In riparian zones and riverine sediments, PAH accumulation is intensified by high OC content, where sorption is influenced by π-π interactions and hydrophobic affinity [213,214]. Duttagupta et al. [191] showed that the mineralogical composition of sediments—especially the dominance of quartz and kaolinite—also determines PAH sorption preferences across different land-use zones.

Desorption—the release of PAHs back into the water column—is influenced by sediment aging, environmental gradients, and physicochemical interactions. Lu et al. [215] demonstrated that sediment aging reduces desorption efficiency due to increased molecular bonding and compaction within sediment matrices. Their kinetic and thermodynamic analyses revealed that desorption follows a three-stage process (fast, slow, and smooth) and is inhibited under strongly acidic/basic pH or high salinity conditions while being promoted by low oxygen and small particle size. Thermal desorption experiments further confirmed that water content and OM reduce PAH release, and that desorption is thermally activated [216]. Additionally, Oyo-Ita et al. [205] reported seasonal variability in salinity and suspended solids as key controls on PAH phase exchange, where higher salinity can inhibit desorption and promote retention in suspended matter. An expansion on these findings showed that both biodegradable and non-biodegradable microplastics sorb and release petroleum hydrocarbons, following reversible pseudo-second-order kinetics [217].

Freshwater chemical conditions—such as pH, redox potential, and ionic strength—also shape PAH behavior by influencing their partitioning and transformation. For instance, Salowsky et al. [73] showed that transient redox conditions in groundwater systems affect PAH retention and biodegradation through variable oxygen availability and iron-reducing microbial processes. Sediments may function as sinks or sources of PAHs depending on molecular structure and surrounding chemical gradients, with fugacity fraction analyses revealing the re-emission of lighter PAHs and the accumulation of heavier congeners in riverine environments [74]. Almouallem et al. [218] demonstrated that increasing ionic strength enhances the sorption of certain PAHs by promoting stronger interactions with sediment particles, whereas low liquid–solid ratios increase adsorption capacity.

In summary, the chemical transport of PAHs in freshwater systems is driven by the interplay of sorption and desorption processes, modulated by sediment composition, OM properties, PAH molecular structure, and dynamic environmental factors such as pH, ionic strength, temperature, and redox potential. Understanding these chemical interactions is essential for predicting the long-term fate and mobility of PAHs in freshwater environments.

#### 3.1.3. Biological Transport Mechanisms

Biological transport mechanisms influence the redistribution of PAHs in freshwater ecosystems through interactions with aquatic organisms and their associated processes. Seasonal algae blooms can enhance the biological pump effect, increasing the transfer of PAHs from the water column to SPM and sediments, especially during high-productivity periods in eutrophic lakes [202,219]. This algae-driven sequestration is often associated with elevated concentrations and toxicity of PAHs in shallow, nutrient-rich environments. Similarly, phytoplankton biomass in hypereutrophic lakes can increase the bioavailability of PAHs in sediments, facilitating their uptake by benthic organisms such as clams and mussels [220]. In cyanobacteria- and macrophyte-dominated systems, the differential distribution of PAHs in riverine versus lacustrine sediments suggests biological community structure influences PAH fate [221]. PAHs can also be actively accumulated and biomagnified through food web interactions, with zooplankton and fish incorporating PAHs in their tissues depending on feeding strategies and habitat [222,223]. Freshwater invertebrates such as gastropods also accumulate PAHs in their tissues, as seen in *S. quadrata*, with levels influenced by local pollution and anthropogenic activity [224]. Moreover, microbial processes—including the downward migration of filamentous bacteria—may enhance vertical PAH migration in sediments [225]. Together, these biological mechanisms interact with hydrological and physicochemical processes to shape the spatiotemporal dynamics and ecological risks associated with PAHs in freshwater systems.

In summary, the transport of PAHs in freshwater systems is governed by a combination of physical, chemical, and biological mechanisms, each modulated by environmental and hydrological forces that shape their mobility, transformation, and persistence. These mechanisms interact within system-specific contexts—such as river flow, sediment composition, water chemistry, and biological activity—producing distinct transport patterns across aquatic compartments. Recognizing the interdependence among these processes is essential to understanding how PAHs are distributed across freshwater landscapes. The following section expands on these dynamics by examining the physicochemical, hydrological, anthropogenic, and natural factors that influence the spatial and temporal distribution of PAHs in freshwater systems.

### 3.2. Factors Affecting Distribution of PAHs

The distribution of PAHs in freshwater systems is influenced by a diverse range of environmental factors, including the physicochemical and hydrological characteristics unique to each system, environmental conditions, such as pH, redox potential, and temperature, anthropogenic activities, and associated pollutant discharges, as well as natural factors like soil composition, land cover, and vegetation. These factors interact in complex ways to influence the distribution, partitioning, and fate of PAHs in freshwater environments. This section explores the interaction among these influences, emphasizing their roles in shaping the spatial distribution of PAHs in freshwater systems.

The distribution of PAHs in freshwater systems is governed by their intrinsic physicochemical properties, such as hydrophobicity, solubility, and volatility, which vary with molecular weight and affect their behavior [72,73,74,75]. Seasonal temperature changes and the presence of OC in sediments lead to fluctuations in PAH concentrations, with higher levels observed during dry periods due to reduced dilution and an increased prevalence of HMW-PAHs under warmer conditions [226,227,228,229]. In lake systems, both external factors (e.g., seasonal salinity, temperature) and internal factors (e.g., sediment characteristics, particulate OC) control PAH partitioning, affecting their distribution among dissolved and particulate phases [29,101,205,230]. Karst springs, wetlands, and transitional estuarine areas display unique hydrological traits that influence PAH distribution, driven by the interplay of hydrodynamics, dilution, and physicochemical properties [139,153,231].

Environmental conditions in water, such as pH, redox potential, ionic strength, and temperature variations, impact PAH behavior in freshwater systems. Extreme pH levels and anaerobic conditions can increase PAH solubility and reduce degradation rates, while higher ionic strength enhances sorption to sediments [73,101,218]. On the other hand, temperature fluctuations affect PAH volatility, solubility, and microbial degradation [116], creating complex distribution patterns that are further intensified by anthropogenic activities.

Anthropogenic activities, including industrial discharges, wastewater and sewage discharges, fuel and coal combustion, vehicle emissions, and oil spills, contribute to PAH pollution, creating localized hotspots that alter their spatial distribution within freshwater systems [96,138,184,186]. These hotspots arise from concentrated pollutant inputs, which vary depending on the type and intensity of human activities in the surrounding region [123]. While nonpoint sources generate diffuse pollution over large areas, their cumulative effects in regions with intense anthropogenic activities often result in localized hotspots [232].

In addition to these localized hotspots, the co-occurrence of other pollutants, such as heavy metals and microplastics, further intensifies the complexity of PAH distribution dynamics. Heavy metals interact with PAHs, forming complexes that alter their solubility and distribution, often resulting in overlapping distribution patterns in hotspots where both pollutants share common anthropogenic sources and environmental processes [233]. Similarly, microplastics act as carriers for PAHs through surface adsorption mechanisms, influencing their distribution, persistence, and bioavailability [234,235,236]. Also, construction activities and regional economic developments near freshwater bodies complicate and alter these distribution dynamics of PAHs by introducing additional contaminants [123,237,238]. Collectively, these anthropogenic influences highlight the complex interaction of pollutants that shape the distribution of PAHs in freshwater systems.

Natural factors, such as soil composition, land cover, and vegetation, shape the distribution and fate of PAHs in freshwater environments. Organic-rich soils enhance PAH adsorption, reducing their mobility and serving as long-term reservoirs for these pollutants [239]. Similarly, riparian vegetation acts as a natural buffer, trapping runoff sediments and promoting microbial degradation of PAHs before reaching water bodies, thereby mitigating their impact [44,191,209,240]. In wetland environments, the interaction between OM and hydrodynamic processes affects the sequestration of PAHs, particularly of HMW-PAHs, within sediment layers [139,241]. Additionally, geological features such as karst formations influence PAH transport by facilitating rapid infiltration and storage in subsurface freshwater environments [86,152,153]. These natural processes interact with environmental conditions, such as seasonal temperature changes and hydrodynamic forces, further influencing PAH partitioning and mobility [74,207,228]. While natural factors often act as mitigating mechanisms, they also highlight the need for comprehensive monitoring strategies to evaluate the interplay between natural and anthropogenic influences on PAH dynamics. Understanding how these factors interact and influence PAH dynamics in freshwater systems requires a detailed examination of their interactions with environmental matrices, such as sediments, water, aquifers, and porewater, which ultimately shape their distribution, bioavailability, and ecological impact.

### 3.3. Interactions with Environmental Matrices

The distribution and fate of PAHs in freshwater systems rely heavily on their interactions with environmental matrices, including sediments, water, aquifers, and porewater. Sediments, serve as a major reservoir for PAHs, especially HMW-PAHs, as observed in stormwater retention ponds and natural lakes [242]. The partitioning of PAHs between sediments and water determines their bioavailability and toxicity to benthic organisms [45,243]. For example, PAH concentrations in the clam *Corbicula fluminea* strongly correlate with sediment PAH levels, indicating sediments as the primary source of bioavailable PAHs [244]. Also, the ability of sediments to act as both a sink and a secondary source of PAHs depends on environmental conditions, such as OM content, total OC levels, and hydrodynamic forces, with steric effects driving the sequestration of HMW-PAHs in fine sediment pores [209]. This mechanism, driven by hydrodynamic forces, resuspends sediment-bound PAHs into the water column, thereby influencing their distribution and ecological impact [123,198]. Consequently, sediment dynamics, including deposition and resuspension, shape the fate and distribution of PAHs in aquatic ecosystems.

In the water phase, PAHs exhibit varying behavior depending on their solubility and hydrophobicity, which govern their partitioning between dissolved and SPM phases [165]. LMW-PAHs, such as NaP, are more soluble and tend to remain in the dissolved phase, while HMW-PAHs, such as BaP, are more likely to adhere to SPM [245]. In the case of resuspension events tend to redistribute sediment-bound PAHs throughout water bodies [74,246]. The properties of SPM, shaped by location, season, and human activities, play a crucial role in PAH distribution by increasing sorption to OC [247]. The solubility and partitioning of PAHs within the water column can serve as predictive factors for their distribution and movement, particularly under the influence of local environmental conditions.

In subsurface environments, aquifers serve as reservoirs for PAHs, with strong adsorption processes in the vadose zone facilitating their retention. The distribution and retention of PAHs in aquifers are primarily governed by their interactions with fine particulate matter and OC, which promote the sequestration of HMW-PAHs [129,248]. This partitioning behavior varies at the sediment–water interface, driven by differences in molecular sizes, with larger PAHs exhibiting higher adsorption potential [249]. Furthermore, minerals such as quartz and kaolinite significantly enhance PAH retention in groundwater systems, highlighting the pivotal role of aquifer geological composition in shaping PAH distribution [191]. Collectively, these processes establish aquifers as essential long-term storage matrices that influence the mobility, fate, and ecological risk of PAHs in freshwater systems.

Porewater, located in the interstitial spaces between sediment particles, is a medium for the distribution of PAHs, enhancing their toxicity to aquatic organisms. LMW-PAHs are often released from sediment-associated OC into porewater, contributing to their bioavailability and toxicity [250]. Toxicological studies conducted in the amphipod *Hyalella azteca* and zebrafish embryos (*Danio rerio*) have demonstrated that PAH-contaminated porewater induces cardiovascular toxicity and decreases survival rates [251]. The freely dissolved fractions of PAHs in porewater, particularly during wet seasons, are readily bioaccumulated by benthic species, resulting in adverse health effects [251].

The interaction of PAHs with environmental matrices, including sediments, water, aquifers, and porewater, shape their distribution and ecological impact in freshwater systems. Sediments function as both a sink and source of PAHs, with processes such as resuspension and partitioning between sediment and water phases influencing their bioavailability and toxicity to aquatic organisms. In the water column, PAH behavior is governed by their physicochemical properties, which determine their partitioning between dissolved and SPM phases. On the other hand, the retention and distribution of PAHs in aquifers and porewater are influenced by interactions with environmental factors such as particulate matter, OC, and minerals. Larger PAH compounds exhibit stronger adsorption in groundwater systems, while freely dissolved PAHs in porewater contribute to toxicity and bioaccumulation in aquatic organisms. Finally, the complex interactions between PAHs and these environmental matrices dictate their long-term distribution, fate, mobility, and ecological risk within freshwater ecosystems.

## 4. Ecotoxicological Impacts of PAHs in Freshwater Systems

### 4.1. Molecular and Cellular Level Effects

**Figure 7:**
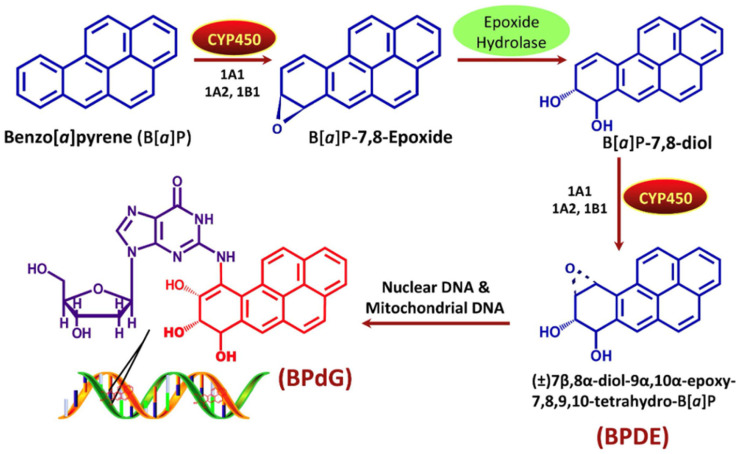
Primary mechanism of DNA binding by BaP, a pro-carcinogenic PAH. Reprinted with permission from ref. [257], under the terms of the Creative Commons Attribution License (CC BY). Copyright 2018, published by Portland Press.

PAHs interact with cellular components and induce oxidative stress (OS) through the generation of reactive oxygen species (ROS), either directly or via metabolic intermediates [252,253,254]. These mechanisms encompass three major pathways—radical-cation, diol-epoxide, and quinone—that contribute to DNA damage [255,256]. As illustrated in Figure 7, BaP undergoes metabolic activation via cytochrome P450 (CYP) enzymes and epoxide hydrolase, forming DNA adducts that promote genotoxicity [257]. This activation leads to oxidative metabolism, where CYP enzymes generate diol-epoxides, o-quinones, and radical cations, each with specific toxic effects [258,259,260]. Diol-epoxides form stable DNA adducts [260], radical cations produce genetic mutations [258], and o-quinones promote OS by generating ROS [261]. These ROS—including superoxide anions (O_2_^−^), hydrogen peroxide (H_2_O_2_), and hydroxyl radicals (∙OH)—overwhelm antioxidant defenses such as glutathione [58], superoxide dismutase [262,263], and catalase [264,265], ultimately causing damage to lipids, proteins [266], and DNA, which triggers apoptosis or necrosis [265,266,267,268].

DNA adduct formation by PAHs is closely associated with mutagenesis and carcinogenesis [269]. BaP is especially potent due to its stable DNA adducts, serving as a model compound for genotoxicity research [270,271]. The mutagenic potential of PAHs depends on their chemical structure and metabolic activation efficiency [253,258,272,273,274]. Furthermore, BaP induces epigenetic changes by altering DNA methylation—either hypo- or hypermethylation—depending on concentration and tissue type, disrupting gene regulation and potentially affecting offspring [275]. BaP also affects the biotin homeostasis pathway and circadian rhythm regulation, as observed in marine medaka, with heritable osteotoxicity in the F3 generation [275]. These effects are mediated by CpG–BPDE adducts and altered DNA methyltransferase and histone deacetylase activity [275]. CYP1A expression, used as a biomarker for PAH exposure, shows strong tissue-specific responses. For example, in *Gambusia affinis*, BaP exposure induces CYP1A mRNA expression in the testis, liver, and other organs, with distinct temporal patterns [276].

PAHs also act as endocrine disruptors by mimicking estrogen or interfering with hormone pathways [48,277]. Their metabolites bind estrogen receptors, altering signaling and disrupting physiological processes such as reproduction and development [278,279,280,281,282,283]. The endocrine-disruptive potential of PAHs varies among different compounds and is mediated by their chemical structures and metabolic products [284]. Additionally, specific PAHs exhibit distinct interactions with hormone receptors, further complicating the effects [253,278]. Endocrine disruption in aquatic species can result in diverse and profound health effects, potentially leading to long-term ecological consequences.

In summary, PAHs impact cellular and molecular functions through mechanisms involving OS, mutagenesis, epigenetic alteration, and endocrine disruption, affecting not only individual organism health but potentially the stability of entire aquatic ecosystems [285].

### 4.2. Impacts on Freshwater Biota

PAH contamination has been widely associated with reproductive and developmental disruptions in aquatic wildlife, often resulting in long-term ecological consequences that may extend across generations [286]. Chronic exposure, even at environmentally relevant concentrations, has been linked to persistent physiological and behavioral impairments in aquatic organisms [48], ranging from sub-lethal effects such as neurobehavioral disruptions [59] to severe tissue damage and mortality. In Caspian White fish (*Rutilus frissi*), exposure to environmentally relevant concentrations of BaP led to significant DNA damage, increased frequency of micronuclei in erythrocytes, and histopathological lesions in liver and gills [287]. Similarly, *Corbicula fluminea*, a native freshwater clam, exhibited PAH accumulation levels that closely correlated with sediment contamination, indicating sediment as a major exposure route through benthic feeding [244]. Experimental oil spills using diluted bitumen (dilbit) in limnocorrals demonstrated that insect emergence declined significantly in a dose-dependent manner, with notable shifts in benthic community structure despite no measurable loss in total abundance [288]. Sublethal effects were also observed in *Hyalella azteca* and *Chironomus riparius* exposed to dilbit-spiked sediments, with amphipods showing higher sensitivity and significant size reduction [289]. BaP has been reported to exhibit LC₅₀ values as low as 5 µg/L for *Daphnia pulex* after 96 h of exposure and 5.6 µg/L for *Pimephales promelas* under UV-enhanced conditions, indicating its high acute toxicity to freshwater organisms [290]. These studies emphasize how PAH-rich unconventional oils compromise the physiological integrity and growth of benthic invertebrates.

Macroinvertebrate assemblages in the Persian Gulf’s Nayband Bay, impacted by oil-related industrial activity, displayed significantly reduced abundance and species richness in stations with high levels of PAHs and total petroleum hydrocarbons (TPHs), revealing strong negative correlations with sedimentary pollutant levels [291]. Similarly, microbial communities in the Pearl River Estuary were disrupted by PAH contamination, which influenced microbial structure, diversity, and deterministic assembly processes across both water and sediment habitats [76]. Comparative studies of microbial communities in lake and river sediments have shown that riverine systems with higher degradation rates of PAHs support natural attenuation, while lacustrine sediments often require biostimulation due to lower functional activity [292]. These disruptions to microbial networks can cascade through the ecosystem, ultimately affecting nutrient cycling, bioremediation capacity, and the health and stability of macroinvertebrate communities.

Taken together, these studies highlight how PAHs exert diverse and sometimes cascading impacts across freshwater biota—affecting microbial assemblages, benthic organisms, and fish—ultimately impairing the structure and function of aquatic communities. These effects are often mediated by key toxicological mechanisms such as bioaccumulation, genotoxicity, mutagenesis, and carcinogenesis, which are further explored in the following subsections.

#### 4.2.1. Bioaccumulation

The hydrophobic and lipophilic nature of PAHs promotes their accumulation in the fatty tissues of aquatic organisms, facilitating bioaccumulation throughout freshwater food webs. In Lake Chaohu (China), PAHs were detected in a variety of freshwater organisms including fish, with significant concentrations observed particularly in lipid-rich tissues, such as the brain and gills; this bioaccumulation posed notable carcinogenic risks to human consumers [272]. In the Ogbese River, Nigeria, shellfish like periwinkles, snails, and mussels exhibited higher biota–sediment accumulation factors compared to fish, with LMW-PAHs dominating the congener profiles [222]. Top predators are not exempt from exposure. In the Um Alnaaj Marsh, Iraq, muscle tissue of waterfowl such as *Anas platyrhynchos*, *A. crecca*, and *A. acuta*, revealed high concentrations of pyrogenic PAHs, suggesting that these birds bioaccumulate contaminants through trophic transfer and present a potential public health concern for consumers of bird meat [293]. In the Pearl River Delta, South China, trophic levels and aquatic productivity were found to significantly influence the distribution and bioconcentration of PAHs in algae and zooplankton, where high chlorophyll-a levels corresponded with greater PAH accumulation [294]. Moreover, a broader analysis in Laizhou Bay demonstrated that algae accumulated the highest concentrations of PAHs, followed by benthic fauna and fish, revealing a biodilution pattern along the food web—though HMW-PAHs still exhibited strong bioaccumulation factors [295].

PAH structure plays a crucial role in bioaccumulation potential. For example, fluoranthene and pyrene, both 3- and 4-ring PAHs, were dominant in the muscle tissues of demersal fish species from the Fengshan River system [234]. PAHs with intermediate log Kow values (5.0–5.6) have been identified as most prone to bioaccumulation [222,296]. A study of 30 rivers in Taiwan also found that up to 99.7% of PAHs bioaccumulated in fish were HMW-PAHs, especially BaP, which poses a substantial carcinogenic risk [103]. These findings collectively demonstrate how trophic level, lipid content, and PAH physicochemical properties influence the extent of bioaccumulation, ultimately compromising food web integrity and the sustainability of freshwater fisheries [297].

#### 4.2.2. Genotoxic, Mutagenic, and Carcinogenic Effects

**Figure 8:**
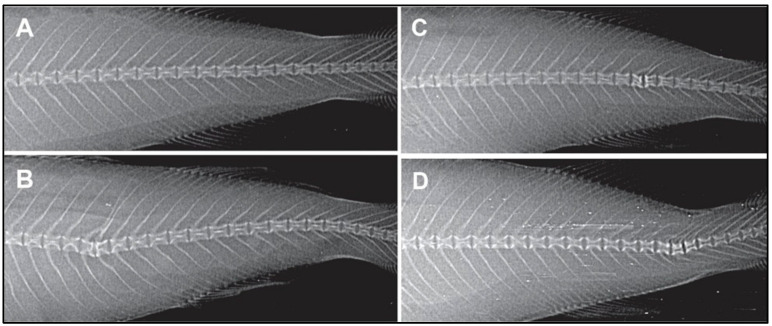
X-ray images of haddock (Melanogrammus aeglefinus) under different treatments: (**A**) control, (**B**) PAH exposure, and (**C**,**D**) oil exposure. The images show various vertebral malformations, including deformities in vertebrae. Reprinted from Meier et al. [57] under the terms of the Creative Commons Attribution License (CC BY). Copyright 2020, published by PLoS ONE.

Several PAHs, especially BaP, are classified as human carcinogens, associated with cancers of the lung, cervix, and prostate [275,298]. Upon metabolic activation by CYP enzymes, particularly CYP1A, BaP forms reactive metabolites, such as BPDE, that bind to DNA, leading to mutations and initiation of carcinogenic processes. Consistent with cellular-level responses, CYP1A was significantly upregulated in the testis, liver, and brain of *Gambusia affinis* following BaP exposure, reflecting systemic genotoxic sensitivity to PAHs in aquatic vertebrates [276]. In juvenile haddock (*Melanogrammus aeglefinus*), both dietary and injected exposure to PAHs led to DNA adduct formation in the liver and intestines, skeletal deformities, and persistent genotoxic effects even after a two-month recovery period (See Figure 8) [57]. Similarly, hepatocellular fibrillar inclusions in European flounder (*Platichthys flesus*) from polluted UK estuaries have been linked to PAH metabolism, particularly in males, suggesting a sex-specific sensitivity to PAH-induced liver pathology [299].

Fish embryos represent another highly sensitive model. Research on teleost fish, including tunas and Japanese medaka (*Oryzias latipes*), revealed that early-stage exposure to PAHs caused developmental abnormalities, especially in cardiac morphology and function, with evidence of phototoxicity and mortality at trace concentrations [300,301,302]. In addition, exposure to NaP reduced acetylcholinesterase activity in the brain, liver, and gills of *Anabas testudineus*, demonstrating clear neurotoxic effects [303]. Other PAHs like phenanthrene and pyrene altered intracellular calcium regulation and suppressed sarco/endoplasmic reticulum Ca²⁺-ATPase (SERCA) gene expression, contributing to cardiotoxicity and muscle impairment [304].

Exposure to PAH mixtures exacerbates toxicity. For example, hepatocyte cultures of *Sparus aurata* exposed to mixtures of phenanthrene, BaP, and benzo[b]fluoranthene exhibited enhanced CYP1A expression and DNA strand breaks compared to single compound exposure, indicating synergistic genotoxicity [305]. In field studies, exposure to Σ16PAHs in vehicle-wash wastewater increased DNA damage in both fish and freshwater mussels, with mussels showing higher genotoxic sensitivity [306]. Similarly, water from the Dnieper River showed mutagenic potential in bioassays despite treatment at municipal plants, highlighting persistent contamination by legacy PAHs [307]. Long-term PAH pollution in Lithuanian rivers and lakes revealed genotoxicity gradients influenced by ecosystem type and hydrodynamics, with lacustrine systems exhibiting higher genotoxic risk but potential biotic adaptation over time [66]. Collectively, these reports underscore the widespread genotoxic, mutagenic, and carcinogenic effects of PAHs across freshwater species, influenced by environmental conditions, mixture composition, and exposure history.

## 5. Conclusions

This comprehensive review has examined the sources, distribution, and ecological impacts of PAHs in freshwater systems. Rivers, streams, lakes, wetlands, groundwater, and glaciers are influenced by a combination of natural and anthropogenic PAH sources. Rivers and streams function as conduits, transporting PAHs from upstream sources, while lakes, particularly in urban areas, accumulate LMW-PAHs in water columns and HMW-PAHs in sediments due to atmospheric deposition and surface runoff. Wetlands are especially vulnerable to wastewater discharge and oil-related activities, leading to significant accumulation of sediment-bound PAHs. On the other hand, groundwater systems face increasing contamination from PAHs leaching through soil and surface water infiltration, with agricultural and industrial regions being particularly at risk. Meanwhile, glaciers are impacted by long-range transported PAHs that deposit in these environments and are subsequently released during glacial melting. Effective monitoring and management strategies are essential across all freshwater systems to mitigate both historical and ongoing sources of PAH pollution.

The distribution of PAHs in freshwater systems is shaped by complex interactions among physical, chemical, biological, and environmental mechanisms. Physical mechanisms, such as advection, diffusion, and sediment transport, drive the movement of PAHs, while chemical mechanisms, including sorption and desorption, determine their mobility and persistence. These chemical mechanisms are further influenced by environmental conditions such as water pH and ionic strength. Biological mechanisms contribute to PAH transport through processes such as bioaccumulation and degradation by aquatic organisms. Additionally, environmental factors, such as temperature, sediment characteristics, and human activities, influence the partitioning and retention of PAHs within various freshwater matrices. These dynamic interactions highlight the complex nature of PAH distribution in freshwater systems, where comprehensive monitoring and management strategies are recommended.

The ecological impacts of PAHs in freshwater systems manifest at the molecular, cellular, and organismal levels, collectively posing significant risks to aquatic biodiversity. At the cellular level, PAHs induce OS, DNA damage, and endocrine disruption, with metabolites such as diol-epoxides forming DNA adducts that increase mutagenic and carcinogenic potential. These disruptions compromise genomic stability and affect reproduction and development in aquatic organisms. Chronic exposure to PAHs exacerbates these issues across freshwater ecosystems, particularly among sensitive species such as invertebrates, fish, and microbial communities. These impacts extend through the food web, with the bioaccumulation of PAHs in aquatic species consumed by humans indicating an urgent need for stringent and ongoing remediation efforts. Given the genotoxic, mutagenic, and carcinogenic properties of PAHs, an ecological approach is essential to understanding and mitigating their effects for the protection of biodiversity and ecosystem function.

In conclusion, this comprehensive review highlights the diverse sources, distribution mechanisms, and ecological impacts of PAHs in freshwater systems, emphasizing the extensive and complex interactions that drive their dynamics and persistence in these environments. PAH dynamics are influenced by local environmental factors and transport mechanisms that promote their persistence in sediments, water, and biota, leading to bioaccumulation and posing significant ecological and health risks to aquatic organisms and humans. The main toxicological effects include molecular and cellular damage, endocrine disruption, genotoxicity, mutagenicity, and carcinogenicity. While substantial progress has been made in understanding these processes, critical gaps remain in quantifying the long-term ecological effects of PAHs across different freshwater environments and species, as well as assessing the influence of climate change on their dynamics. Addressing these gaps through more comprehensive, ecosystem-level studies is essential for advancing PAH pollution research, management, and remediation strategies. These efforts should include monitoring across diverse environmental matrices, developing advanced and rapid analytical methodologies, and establishing robust regulatory frameworks that incorporate input from diverse stakeholders. A collaborative approach that integrates scientific research with policy action is vital to protecting and sustaining freshwater ecosystems for future generations.

## Figures and Tables

**Table 1:** Summary of PAH contamination in selected freshwater systems (rivers and lakes).

River System (Region)	Main PAHs Detected	Sources	Ecological Effects	References
Pearl River (China)	Fluoranthene, Pyrene, Benzo[a]pyrene	Industrial discharges, urban runoff	Bioaccumulation in fish; risk to benthic fauna	[125]
Yangtze River (China)	Phenanthrene, Fluoranthene, Chrysene	Domestic sewage, agricultural runoff	Chronic exposure in mollusks; endocrine disruption	[126]
Tigris River (Iraq)	Benzo[a]anthracene, Chrysene	Wastewater discharges, oil refinery pollution	Decreased diversity of benthic invertebrates; moderate ecological risk	[127]
Ogun River (Nigeria)	Naphthalene, Acenaphthene	Industrial effluents	Risk to biota	[128]
Taihu Lake (China)	Phenanthrene, Pyrene, Benzo[a]anthracene	Urban-industrial interface	Oxidative stress in fish and invertebrates	[129]
Chaohu Lake (China)	Fluoranthene, Pyrene, Benzo[a]anthracene	Industrial discharges, urban runoff, atmospheric deposition	Moderate ecological risk; potential impact on aquatic organisms	[130]

## Data Availability

This study is a review article, and all data analyzed or cited are derived from publicly available studies and sources, as referenced within the manuscript. No new datasets were generated for this study. Any additional information can be obtained from the corresponding author upon reasonable request.

## Supplementary Materials

The following supporting information can be downloaded at https://www.mdpi.com/article/10.3390/toxics13040321/s1, Table S1: Physical properties and chemical structure of the 16 USEPA priority PAHs; Table S2: Origin and sources of PAHs in various types of freshwater systems.

## Abbreviations

The following abbreviations are used in this manuscript:

PAHs	Polycyclic aromatic hydrocarbons
LMW-PAHs	Low molecular weight polycyclic aromatic hydrocarbons
HMW-PAH	High molecular weight polycyclic aromatic hydrocarbons
LRTs	Long-range transport
USEPA	United States Environmental Protection Agency
WHO	World Health Organization
SPM	Suspended particulate
OM	Organic matter
OC	Organic carbon
BaP	Benzo[a]pyrene
ROS	Reactive oxygen species
OS	Oxidative stress
CYP	Cytochrome P450
Log Kow	Octanol-water partitioning
