# Supplementary material for: Polycyclic Aromatic Hydrocarbons (PAHs) in Freshwater Systems: A Comprehensive Review of Sources, Distribution, and Ecotoxicological Impacts
Source: Toxics. 2025 Apr 20;13(4):321. doi: 10.3390/toxics13040321 (PMC12031217; doi:10.3390/toxics13040321)
Supplement: Supplementary file 1 [file toxics-13-00321-s001.zip › toxics-3540926-supplementary.pdf]

toxics MDPI

Review

Polycyclic Aromatic Hydrocarbons (PAHs) in Freshwater Systems: A Comprehensive Review of Sources, Distribution, and Impacts

Pedro J. Berríos-Rolón, María C. Cotto*, and Francisco Márquez

Nanomaterials Research Group, Department of Natural Sciences and Technology, Division of Natural Sciences, Technology and Environment, Universidad Ana G. Méndez-Gurabo Campus, 00778PR, United States; berriosp1@uagm.edu (P.J.B.-R.); mcotto48@uagm.edu (M.C.); fmarquez@uagm.edu (F.M.)

* Correspondence: mcotto48@uagm.edu (MC), Tel.: +1-787-743-7979 (ext. 4491)

Supplementary Materials

Contents

Table S1. Physical Properties And Chemical Structure Of The 16 Usepa Priority PAHs. 1

Table S2. Origin And Sources Of PAHs In Various Types Of Freshwater Systems. 3

References 7

toxics

MDPI

Table S1. Physical properties and chemical structure of the 16 USEPA priority PAHs.

No.	Name	CS[a]	Rings	Class.[b]	MW[c]	BP[d]	MP[e]	S[f]	Log K_{ow}[g]	VP[h]
1	Naphthalene	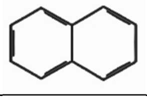	2	LMW	128.17	209	80	31.0	3.37	$8.89E^{-2}$
2	Acenaphthylene	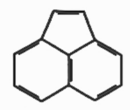	3	LMW	152.19	290	124	16.1	4.00	$2.90E^{-0.2}$
3	Acenaphthene	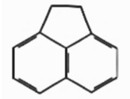	3	LMW	154.21	252	108	3.8	3.92	$3.75E^{-3}$
4	Fluorene	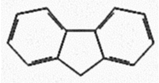	3	LMW	166.22	276	119	1.9	4.18	$3.24E^{-3}$
5	Phenanthrene	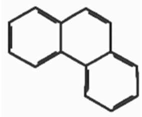	3	LMW	178.23	326	136	1.1	4.57	$6.80E^{-4}$
6	Anthracene	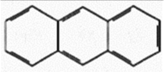	3	LMW	178.23	326	136	0.045	4.54	$2.55E^{-5}$
7	Fluoranthene	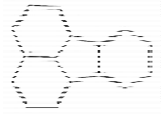	4	HMW	202.25	369	166	0.26	5.22	$8.13E^{-6}$
8	Pyrene	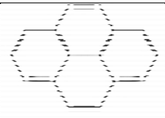	4	HMW	202.25	369	166	0.132	5.18	$4.25E^{-6}$
9	Benzo[a]anthracene	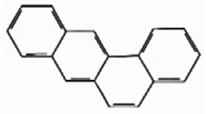	4	HMW	228.30	400	177	0.011	5.91	$1.54E^{-7}$
10	Chrysene	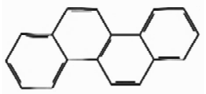	4	HMW	228.30	400	177	0.0015	5.91	$7.80E^{-9}$
11	Benzo[b]fluoranthene	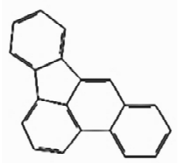	5	HMW	252.30	461	209	0.0015	5.80	$8.06E^{-8}$
12	Benzo[k]fluoranthene	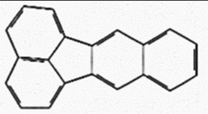	5	HMW	252.30	430	194	0.0008	6.00	$9.59E^{-11}$
13	Benzo[a]pyrene	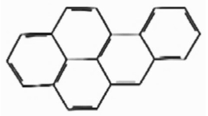	5	HMW	252.30	461	209	0.0038	5.91	$4.89E^{-9}$

14	Indeno[1,2,3-cd]pyrene	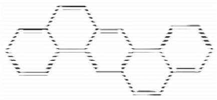	6	HMW	276.30	498	233	0.062	6.50	1.40E^{-10}
15	Dibenz[a,h]anthracene	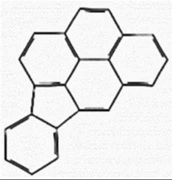	6	HMW	278.30	487	218	0.0005	6.75	2.10E^{-11}
16	Benzo[g,h,i]perylene	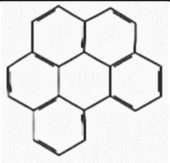	6	HMW	276.30	467	218	0.00026	6.50	1.00E^{-10}

(a) CS: Chemical structure [1]; (b) Classification as LMW-PAHs and HMW-PAHs; (c) MW: Molecular weight in g/mol [1]; (d) BP: Boiling point in °C [2]; (e) MP: Melting point in °C [2]; (f) S: Water solubility at 25°C [3]; (g) Log K_{ow}: Octanol water partitioning [4]; (h) VP: Vapor pressure mmHg at 25°C [3].

Table S2. Origin and sources of PAHs in various types of freshwater systems.

Freshwater system	Site name and Location	Environmental matrix	PAH Origin	PAH source	Reference
lake	Tangxun and Dong Lake, Wuhan, China	water	mixed	Petroleum<br>biomass<br>heavy oils<br>natural gas	[5]
lake	Soltaire and Fairbank, Central Ontario, Canada	sediment	pyrogenic	particulate deposition<br>grass<br>wood<br>gasoline<br>coal	[6]
lake	Shangai, China	sediment	pyrogenic	vehicle emission<br>coal<br>biomass<br>natural gas combustion	[7]
lake	Lake Baikal, Siberia	aerosol	pyrogenic	wildfires	[8]
lake	Lake Baikal, Russia	water<br>aerosol	pyrogenic	wildfires	[9]
river	Jiulong River watershed and estuary, southeast China	water	pyrogenic	fossil fuel combustion<br>petroleum combustion<br>biomass combustion	[10]
river	Rur River catchment, North Rhine-West-phalia, Germany	water<br>sediment	petrogenic	wastewater	[11]
river	Liujiang River Basin, China	sediment	pyrogenic	coal<br>wood<br>weed<br>petroleum<br>gasoline<br>kerosene<br>crude oil	[12]
river	Buffalo River Estuary, South Africa	water<br>sediment	pyrogenic	highway runoff<br>biomass combustion<br>domestic wastes<br>stormwater runoff	[13]
river	Nun River, Bayelsa State, Nigeria	water	petrogenic	oil-related activities	[14]
river	Ekulu, Enugu metropolis, Nigeria	water	mixed	agricultural waste burning<br>vehicular emissions<br>power generators	[15]

river	Euphrates River system, Iraq	water<br>sediment	pyrogenic	petroleum combustion<br>vehicular emissions<br>fossil fuel burning<br>illegal waste disposal<br>wastewater discharge	[16]
river	Huaihe River, China	soil	pyrogenic	vehicular emissions<br>biomass combustion<br>coal combustion	[17]
river	River Benue, Nigeria	sediment	mixed	coal combustion<br>crude oil<br>wood burning<br>vehicular emissions	[18]
river	Yangtze River, China	water<br>sediment	pyrogenic	coal burning<br>coke plants<br>biomass<br>vehicular emissions<br>energy consumption	[19]
river	River systems, Taiwan	sediment<br>fish	pyrogenic	petroleum<br>grass and wood burning<br>coal	[20]
river	Ulhas River, India	sediment	pyrogenic	wood burning<br>coke plants<br>coal<br>gasoline and diesel	[21]
river	Pitimbu River, Natal, Brazil	sediment	pyrogenic	biomass<br>coal<br>petroleum<br>refined oil products	[22]
river	Eastern Tibetean Plateau, China	water	pyrogenic	coal<br>charcoal<br>coking discharge	[23]
river	Great Lakes tributaries, USA and Canada	sediment	petrogenic	coal-tar-sealed pavement	[24]
stream	Suzhou Industrial Park, China	sediment	pyrogenic	biomass<br>coal<br>petroleum	[25]
stream	Oliwski and Strzyza, Gdansk, Poland	sediment	pyrogenic	biomass<br>coal<br>petroleum	[26]
stream	North-central Portugal	water	pyrogenic	biomass<br>wildfires	[27]

stream	Monastir Bay, Tunisia	sediment	mixed	road runoff<br>domestic wastewater<br>industrial wastewater<br>workshops waste oil<br>industrial activity	[28]
groundwater	Yellow River Estuary, China	water	pyrogenic	fossil fuels<br>coal<br>natural gas<br>vehicular emissions	[29]
groundwater	Chongqing, Southwest, China	water<br>soil	pyrogenic	grass<br>wood<br>coal	[30]
groundwater	Abou Ali River-North Lebanon	water	pyrogenic	fuel<br>incineration<br>miscellaneous burning	[31]
groundwater	Peri-Urban Forest Area, Braga Region, NW Portugal	water	pyrogenic	grass<br>wood<br>coal	[32]
groundwater	Banja Luka, Republic of Srpska, Bosnia and Herzegovina	soil	mixed	traffic emissions<br>coal combustion<br>biomass combustion	[33]
groundwater	Nanchuan catchment, Chongqing, Southwest, China	soil<br>water	pyrogenic	coal combustion<br>vehicular emissions	[34]
groundwater	Lanzhou, Shijiazhuang, and Golmud and Du’an County, China	water	mixed	oil pipeline leakage<br>coal combustion<br>wood and straw burning	[35]
groundwater	Campania Plain, South Italy	water	pyrogenic	carbon and fuel	[36]
groundwater	Eleme, Nigeria	water<br>soil	mixed	petroleum extraction	[37]
wetland	Shadegan wetland, Iran	sediment<br>water	mixed	bulrush burning<br>biomass combustion<br>heavy gasoline oils<br>lubricating oils<br>used motor oils	[38]
wetland	Anzali Wetland, Caspian Sea, Iran	sediment	petrogenic	fuel oil<br>light refined oil	[39]
wetland	Anzali Wetland, Caspian Sea, Iran	water<br>sediment<br>fish	mixed	crude oil combustion<br>diesel fuel<br>biomass<br>coal and oil leakage	[40]

wetland	Coastal Industrial Zone, Tianjin, China	sediment<br>water<br>reed	mixed	fossil fuels combustion<br>petroleum leakage	[41]
wetland	Ashtamudi Wetland, south-west coast, India	sediment	mixed	biomass burning<br>fuel combustion<br>oil leakage	[42]
wetland	Hoor Al-Azim Wetland, Iran	sediment	mixed	fossil fuel combustion<br>oil leakage	[43]
wetland	Momoge Wetland, China	soils	mixed	petroleum combustion<br>coal combustion<br>petroleum spills	[44]
wetland	Miankaleh International Wetland, Iran	water<br>sediment	mixed	fossil fuel<br>sewage discharge<br>port activity	[45]
wetland	Baghjan, upper Assam, India	water<br>sediment<br>fish	petrogenic	oil spill	[46]
peatland	Songkhla Lake Basin Swamp Forest, Thailand	air	pyrogenic	biomass and peat burning	[47]
bog	Southern Taiga of Western Siberia	water<br>peat	pyrogenic	biomass and peat burning<br>wildfires	[48]
marsh	Pantanal Biome, Brazil	soil	pyrogenic	biomass burning<br>wildfires	[49]
glacier	Tibetan Plateau, China	meltwater	pyrogenic	coal incomplete combustion<br>biomass combustion<br>coking discharge	[23]
glacier	Kongsfjorden, Svalbard, Norway	water	pyrogenic	diesel fuel leakage<br>heat and power generation<br>dust from coal mining<br>long-range transport	[50]
glacier	Kongsfjorden, Arctic	water	pyrogenic	grass<br>wood<br>coal	[51]
glacier	Admiralty Bay, King George Island, Maritime Antarctica	water	petrogenic	fuel consumption<br>local human activity	[52]
glacier	King George Island, Antarctica	soil	petrogenic	electricity generators<br>light-duty gasoline<br>fuel consumption	[53]
glacier	Eastern Tibetean Plateau, China	water	pyrogenic	coal<br>charcoal<br>coking discharge	[23]

References

1. National Center for Biotechnology Information PubChem Dataset 2023.
2. Shen, H. *Polycyclic Aromatic Hydrocarbons*; Springer Theses; Springer Berlin Heidelberg: Berlin, Heidelberg, 2016; ISBN 978-3-662-49678-7.
3. Agency for Toxic Substances and Disease Registry Toxicological Profile for Polycyclic Aromatic Hydrocarbons 1996.
4. Mackay, D.; Shiu, W.-Y.; Shiu, W.-Y.; Lee, S.C. *Handbook of Physical-Chemical Properties and Environmental Fate for Organic Chemicals*; 0 ed.; CRC Press, 2006; ISBN 978-0-429-15007-4.
5. Yao, K.; Xie, Z.; Zhi, L.; Wang, Z.; Qu, C. Polycyclic Aromatic Hydrocarbons in the Water Bodies of Dong Lake and Tangxun Lake, China: Spatial Distribution, Potential Sources and Risk Assessment. *Water* **2023**, *15*, 2416, doi:10.3390/w15132416.
6. Colby, G.A. Deposition of Polycyclic Aromatic Hydrocarbons (PAHs) into Northern Ontario Lake Sediments. *bioRxiv* **2019**, 786913, doi:10.1101/786913.
7. Yang, J.; Yang, Y.; Chen, R.-S.; Meng, X.-Z.; Xu, J.; Qadeer, A.; Liu, M. Modeling and Evaluating Spatial Variation of Polycyclic Aromatic Hydrocarbons in Urban Lake Surface Sediments in Shanghai. *Environmental Pollution* **2018**, *235*, 1–10, doi:10.1016/j.envpol.2017.12.032.
8. Golobokova, L.; Khodzher, T.; Khuriganova, O.; Marinayte, I.; Onishchuk, N.; Rusanova, P.; Potemkin, V. Variability of Chemical Properties of the Atmospheric Aerosol above Lake Baikal during Large Wildfires in Siberia. *Atmosphere* **2020**, *11*, 1230, doi:10.3390/atmos11111230.
9. Gorshkov, A.G.; Izosimova, O.N.; Kustova, O.V.; Marinaite, I.I.; Galachyants, Y.P.; Sinyukovich, V.N.; Khodzher, T.V. Wildfires as a Source of PAHs in Surface Waters of Background Areas (Lake Baikal, Russia). *Water* **2021**, *13*, 2636, doi:10.3390/w13192636.
10. Wu, Y.; Wang, X.; Ya, M.; Li, Y.; Hong, H. Seasonal Variation and Spatial Transport of Polycyclic Aromatic Hydrocarbons in Water of the Subtropical Jiulong River Watershed and Estuary, Southeast China. *Chemosphere* **2019**, *234*, 215–223, doi:10.1016/j.chemosphere.2019.06.067.
11. Schwanen, C.A.; Kronsbein, P.M.; Balik, B.; Schwarzbauer, J. Dynamic Transport and Distribution of Organic Pollutants in Water and Sediments of the Rur River. *Water Air Soil Pollut* **2023**, *235*, 9, doi:10.1007/s11270-023-06786-8.
12. Miao, X.; Hao, Y.; Cai, J.; Xie, Y.; Zhang, J. The Distribution, Sources and Health Risk of Polycyclic Aromatic Hydrocarbons (PAHs) in Sediments of Liujiang River Basin: A Field Study in Typical Karstic River. *Marine Pollution Bulletin* **2023**, *188*, 114666, doi:10.1016/j.marpolbul.2023.114666.
13. Adeniji, A.O.; Okoh, O.O.; Okoh, A.I. Levels of Polycyclic Aromatic Hydrocarbons in the Water and Sediment of Buffalo River Estuary, South Africa and Their Health Risk Assessment. *Arch Environ Contam Toxicol* **2019**, *76*, 657–669, doi:10.1007/s00244-019-00617-w.
14. Leizou, K.E.; Ashraf, M.A. Distribution, Compositional Pattern and Potential to Human Exposure of PAHs in Water, Amassoma Axis, Nun River, Bayelsa State, Nigeria. *Acta Chemica Malaysia* **2019**, *3*, 16–20, doi:10.2478/acmy-2019-0002.
15. Umeh, C.T.; Nduka, J.K.; Omokpariola, D.O.; Morah, J.E.; Mmaduakor, E.C.; Okoye, N.H.; Lilian, E.-E.I.; Kalu, I.F. Ecological Pollution and Health Risk Monitoring Assessment of Polycyclic Aromatic Hydrocarbons and Heavy Metals in Surface Water, Southeastern Nigeria. *Environ Anal Health Toxicol* **2023**, *38*, doi:10.5620/eaht.2023007.
16. Grmasha, R.A.; Abdulameer, M.H.; Stenger-Kovács, C.; Al-sareji, O.J.; Al-Gazali, Z.; Al-Juboori, R.A.; Meiczinger, M.; Hashim, K.S. Polycyclic Aromatic Hydrocarbons in the Surface Water and Sediment along Euphrates River System: Occurrence, Sources, Ecological and Health Risk Assessment. *Marine Pollution Bulletin* **2023**, *187*, 114568, doi:10.1016/j.marpolbul.2022.114568.

17. Yuan, Z.; Shi, S.; Wu, X.; Wang, Q.; Wang, S.; Fan, Z. Polycyclic Aromatic Hydrocarbons (PAHs) in Riparian Soils of the Middle Reach of Huaihe River: A Typical Coal Mining Area in China. *Soil and Sediment Contamination: An International Journal* **2022**, *0*, 1–15, doi:10.1080/15320383.2022.2074370.
18. Arowojolu, I.M.; Tongu, S.M.; Itodo, A.U.; Sodre, F.F.; Kyenge, B.A.; Nwankwo, R.C. Investigation of Sources, Ecological and Health Risks of Sedimentary Polycyclic Aromatic Hydrocarbons in River Benue, Nigeria. *Environmental Technology & Innovation* **2021**, *22*, 101457, doi:10.1016/j.eti.2021.101457.
19. Zhao, Z.; Gong, X.; Zhang, L.; Jin, M.; Cai, Y.; Wang, X. Riverine Transport and Water-Sediment Exchange of Polycyclic Aromatic Hydrocarbons (PAHs) along the Middle-Lower Yangtze River, China. *Journal of Hazardous Materials* **2021**, *403*, 123973, doi:10.1016/j.jhazmat.2020.123973.
20. Lee, C.-C.; Chen, C.S.; Wang, Z.-X.; Tien, C.-J. Polycyclic Aromatic Hydrocarbons in 30 River Ecosystems, Taiwan: Sources, and Ecological and Human Health Risks. *Science of The Total Environment* **2021**, *795*, 148867, doi:10.1016/j.scitotenv.2021.148867.
21. Nair, M.M.; Sreeraj, M.K.; Rakesh, P.S.; Thomas, J.K.; Kharat, P.Y.; Sukumaran, S. Distribution, Source and Potential Biological Impacts of Polycyclic Aromatic Hydrocarbons in the Core Sediments of a Networked Aquatic System in the Northwest Coast of India – A Special Focus on Thane Creek Flamingo Sanctuary (Ramsar Site). *Regional Studies in Marine Science* **2024**, 103377, doi:10.1016/j.rsma.2024.103377.
22. dos Santos, P.R.S.; Moreira, L.F.F.; Moraes, E.P.; de Farias, M.F.; Domingos, Y.S. Traffic-Related Polycyclic Aromatic Hydrocarbons (PAHs) Occurrence in a Tropical Environment. *Environ Geochem Health* **2021**, *43*, 4577–4587, doi:10.1007/s10653-021-00947-6.
23. Liu, X.; Dong, Z.; Baccolo, G.; Gao, W.; Li, Q.; Wei, T.; Qin, X. Distribution, Composition and Risk Assessment of PAHs and PCBs in Cryospheric Watersheds of the Eastern Tibetan Plateau. *Science of The Total Environment* **2023**, *890*, 164234, doi:10.1016/j.scitotenv.2023.164234.
24. Baldwin, A.K.; Corsi, S.R.; Oliver, S.K.; Lenaker, P.L.; Nott, M.A.; Mills, M.A.; Norris, G.A.; Paatero, P. Primary Sources of Polycyclic Aromatic Hydrocarbons to Streambed Sediment in Great Lakes Tributaries Using Multiple Lines of Evidence. *Environmental Toxicology and Chemistry* **2020**, *39*, 1392–1408, doi:10.1002/etc.4727.
25. Yuan, Z.; He, B.; Wu, X.; Simonich, S.L.M.; Liu, H.; Fu, J.; Chen, A.; Liu, H.; Wang, Q. Polycyclic Aromatic Hydrocarbons (PAHs) in Urban Stream Sediments of Suzhou Industrial Park, an Emerging Eco-Industrial Park in China: Occurrence, Sources and Potential Risk. *Ecotoxicology and Environmental Safety* **2021**, *214*, 112095, doi:10.1016/j.ecoenv.2021.112095.
26. Nawrot, N.; Pouch, A.; Matej-Łukowicz, K.; Pazdro, K.; Mohsin, M.; Rezania, S.; Wojciechowska, E. A Multi-Criteria Approach to Investigate Spatial Distribution, Sources, and the Potential Toxicological Effect of Polycyclic Aromatic Hydrocarbons (PAHs) in Sediments of Urban Retention Tanks. *Environ Sci Pollut Res* **2023**, *30*, 27895–27911, doi:10.1007/s11356-022-24168-1.
27. Carvalho, F.; Pradhan, A.; Abrantes, N.; Campos, I.; Keizer, J.J.; Cássio, F.; Pascoal, C. Wildfire Impacts on Freshwater Detrital Food Webs Depend on Runoff Load, Exposure Time and Burnt Forest Type. *Science of The Total Environment* **2019**, *692*, 691–700, doi:10.1016/j.scitotenv.2019.07.265.
28. Khiari, N.; Charef, A.; Atoui, A.; Azouzi, R.; Khalil, N.; Khadhar, S. Southern Mediterranean Coast Pollution: Long-Term Assessment and Evolution of PAH Pollutants in Monastir Bay (Tunisia). *Marine Pollution Bulletin* **2021**, *167*, 112268, doi:10.1016/j.marpolbul.2021.112268.
29. Li, J.; Li, F.; Liu, Q. PAHs Behavior in Surface Water and Groundwater of the Yellow River Estuary: Evidence from Isotopes and Hydrochemistry. *Chemosphere* **2017**, *178*, 143–153, doi:10.1016/j.chemosphere.2017.03.052.
30. Sun, Y.; Zhang, S.; Lan, J.; Xie, Z.; Pu, J.; Yuan, D.; Yang, H.; Xing, B. Vertical Migration from Surface Soils to Groundwater and Source Appointment of Polycyclic Aromatic Hydrocarbons in Epikarst Spring Systems, Southwest China. *Chemosphere* **2019**, *230*, 616–627, doi:10.1016/j.chemosphere.2019.05.007.

31. Jabali, Y.; Iaaly, A.; Millet, M. Environmental Occurrence, Spatial Distribution, and Source Identification of PAHs in Surface and Groundwater Samples of Abou Ali River-North Lebanon. *Environmental Monitoring and Assessment* **2021**, *193*, doi:10.1007/s10661-021-09513-0.
32. Mansilha, C.; Melo, A.; Martins, Z.E.; Ferreira, I.M.P.L.V.O.; Pereira, A.M.; Espinha Marques, J. Wildfire Effects on Groundwater Quality from Springs Connected to Small Public Supply Systems in a Peri-Urban Forest Area (Braga Region, NW Portugal). *Water* **2020**, *12*, 1146, doi:10.3390/w12041146.
33. Ilić, P.; Nešković Markić, D.; Stojanović Bjelić, L. Evaluation of Sources and Ecological Risk of PAHs in Different Layers of Soil and Groundwater. **2020**, doi:10.20944/preprints202002.0224.v2.
34. Qi, X.; Lan, J.; Sun, Y.; Wang, S.; Liu, L.; Wang, J.; Long, Q.; Huang, M.; Yue, K. Linking PAHs Concentration, Risk to PAHs Source Shift in Soil and Water in Epikarst Spring Systems, Southwest China. *Ecotoxicology and Environmental Safety* **2023**, *264*, 115465, doi:10.1016/j.ecoenv.2023.115465.
35. Qiao, X.; Zheng, B.; Li, X.; Zhao, X.; Dionysiou, D.D.; Liu, Y. Influencing Factors and Health Risk Assessment of Polycyclic Aromatic Hydrocarbons in Groundwater in China. *Journal of Hazardous Materials* **2021**, *402*, 123419, doi:10.1016/j.jhazmat.2020.123419.
36. Montuori, P.; De Rosa, E.; Cerino, P.; Pizzolante, A.; Nicodemo, F.; Gallo, A.; Rofrano, G.; De Vita, S.; Limone, A.; Triassi, M. Estimation of Polycyclic Aromatic Hydrocarbons in Groundwater from Campania Plain: Spatial Distribution, Source Attribution and Health Cancer Risk Evaluation. *Toxics* **2023**, *11*, 435, doi:10.3390/toxics11050435.
37. Edet, A.; Nyong, E.; Ukpong, A.; Edet, C. Evaluation and Risk Assessment of Polycyclic Aromatic Hydrocarbons in Groundwater and Soil near a Petroleum Distribution Pipeline Spill Site, Eleme, Nigeria. *Sustainable Water Resources Management* **2021**, *7*, doi:10.1007/s40899-021-00530-y.
38. Ashayeri, N.Y.; Keshavarzi, B.; Moore, F.; Kersten, M.; Yazdi, M.; Lahijanzadeh, A.R. Presence of Polycyclic Aromatic Hydrocarbons in Sediments and Surface Water from Shadegan Wetland – Iran: A Focus on Source Apportionment, Human and Ecological Risk Assessment and Sediment-Water Exchange. *Ecotoxicology and Environmental Safety* **2018**, *148*, 1054–1066, doi:10.1016/j.ecoenv.2017.11.055.
39. Yancheshmeh, R.A.; Bakhtiari, A.R.; Mortazavi, S.; Savabieasfahani, M. Sediment PAH: Contrasting Levels in the Caspian Sea and Anzali Wetland. *Marine Pollution Bulletin* **2014**, *84*, 391–400, doi:10.1016/j.marpolbul.2014.05.001.
40. Cheshmvahm, H.; Keshavarzi, B.; Moore, F.; Zarei, M.; Esmaeili, H.R.; Hooda, P.S. Investigation of the Concentration, Origin and Health Effects of PAHs in the Anzali Wetland: The Most Important Coastal Freshwater Wetland of Iran. *Marine Pollution Bulletin* **2023**, *193*, 115191, doi:10.1016/j.marpolbul.2023.115191.
41. Chen, Z.; Ren, G.; Ma, X.; Zhou, B.; Yuan, D.; Liu, H.; Wei, Z. Presence of Polycyclic Aromatic Hydrocarbons among Multi-Media in a Typical Constructed Wetland Located in the Coastal Industrial Zone, Tianjin, China: Occurrence Characteristics, Source Apportionment and Model Simulation. *Science of The Total Environment* **2021**, *800*, 149601, doi:10.1016/j.scitotenv.2021.149601.
42. Sreedevi, M.A.; Harikumar, P.S. Occurrence, Distribution, and Ecological Risk of Heavy Metals and Persistent Organic Pollutants (OCPs, PCBs, and PAHs) in Surface Sediments of the Ashtamudi Wetland, South-West Coast of India. *Regional Studies in Marine Science* **2023**, *64*, 103044, doi:10.1016/j.rsma.2023.103044.
43. Sheikh Fakhradini, S.; Moore, F.; Keshavarzi, B.; Lahijanzadeh, A. Polycyclic Aromatic Hydrocarbons (PAHs) in Water and Sediment of Hoor Al-Azim Wetland, Iran: A Focus on Source Apportionment, Environmental Risk Assessment, and Sediment-Water Partitioning. *Environ Monit Assess* **2019**, *191*, 233, doi:10.1007/s10661-019-7360-0.
44. Xu, J.; Wang, H.; Sheng, L.; Liu, X.; Zheng, X. Distribution Characteristics and Risk Assessment of Polycyclic Aromatic Hydrocarbons in the Momoge Wetland, China. *IJERPH* **2017**, *14*, 85, doi:10.3390/ijerph14010085.
45. Rokhbar, M.; Keshavarzi, B.; Moore, F.; Zarei, M.; Hooda, P.S.; Risk, M.J. Occurrence and Source of PAHs in Miankaleh International Wetland in Iran. *Chemosphere* **2023**, *321*, 138140, doi:10.1016/j.chemosphere.2023.138140.

46. Singh, V.; Negi, R.; Jacob, M.; Gayathri, A.; Rokade, A.; Sarma, H.; Kalita, J.; Tasfia, S.T.; Bharti, R.; Wakid, A.; et al. Polycyclic Aromatic Hydrocarbons (PAHs) in Aquatic Ecosystem Exposed to the 2020 Baghjan Oil Spill in Upper Assam, India: Short-Term Toxicity and Ecological Risk Assessment. *PLoS One* **2023**, *18*, e0293601, doi:10.1371/journal.pone.0293601.
47. Nim, N.; Morris, J.; Tekasakul, P.; Dejchanchaiwong, R. Fine and Ultrafine Particle Emission Factors and New Diagnostic Ratios of PAHs for Peat Swamp Forest Fires. *Environmental Pollution* **2023**, *335*, 122237, doi:10.1016/j.envpol.2023.122237.
48. Russkikh, I.V.; Strel'nikova, E.B.; Serebrennikova, O.V.; Voistinova, E.S.; Kharanzhevskaya, Yu.A. Identification of Hydrocarbons in the Waters of Raised Bogs in the Southern Taiga of Western Siberia. *Geochem. Int.* **2020**, *58*, 447–455, doi:10.1134/S0016702920040072.
49. Caumo, S.; Lázaro, W.L.; Sobreira Oliveira, E.; Beringui, K.; Gioda, A.; Massone, C.G.; Carreira, R.; de Freitas, D.S.; Ignacio, A.R.A.; Hacon, S. Human Risk Assessment of Ash Soil after 2020 Wildfires in Pantanal Biome (Brazil). *Air Qual Atmos Health* **2022**, *15*, 2239–2254, doi:10.1007/s11869-022-01248-2.
50. Ademollo, N.; Spataro, F.; Rauseo, J.; Pescatore, T.; Fattorini, N.; Valsecchi, S.; Polesello, S.; Patrolecco, L. Occurrence, Distribution and Pollution Pattern of Legacy and Emerging Organic Pollutants in Surface Water of the Kongsfjorden (Svalbard, Norway): Environmental Contamination, Seasonal Trend and Climate Change. *Marine Pollution Bulletin* **2021**, *163*, 111900, doi:10.1016/j.marpolbul.2020.111900.
51. Li, R.; Gao, H.; Ji, Z.; Jin, S.; Ge, L.; Zong, H.; Jiao, L.; Zhang, Z.; Na, G. Distribution and Sources of Polycyclic Aromatic Hydrocarbons in the Water Column of Kongsfjorden, Arctic. *Journal of Environmental Sciences* **2020**, *97*, 186–193, doi:10.1016/j.jes.2020.04.024.
52. Szopińska, M.; Szumińska, D.; Bialik, R.J.; Dymerski, T.; Rosenberg, E.; Polkowska, Ż. Determination of Polycyclic Aromatic Hydrocarbons (PAHs) and Other Organic Pollutants in Freshwaters on the Western Shore of Admiralty Bay (King George Island, Maritime Antarctica). *Environ Sci Pollut Res* **2019**, *26*, 18143–18161, doi:10.1007/s11356-019-05045-w.
53. Deelaman, W.; Pongpiachan, S.; Tipmanee, D.; Suttinun, O.; Choochuay, C.; Iadtem, N.; Charoenkalunyuta, T.; Promdee, K. Source Apportionment of Polycyclic Aromatic Hydrocarbons in the Terrestrial Soils of King George Island, Antarctica. *Journal of South American Earth Sciences* **2020**, *104*, 102832, doi:10.1016/j.jsames.2020.102832.
